# Uncovering Disease Mechanisms in a Novel Mouse Model Expressing Humanized APOEε4 and Trem2*R47H

**DOI:** 10.3389/fnagi.2021.735524

**Published:** 2021-10-11

**Authors:** Kevin P. Kotredes, Adrian Oblak, Ravi S. Pandey, Peter Bor-Chian Lin, Dylan Garceau, Harriet Williams, Asli Uyar, Rita O’Rourke, Sarah O’Rourke, Cynthia Ingraham, Daria Bednarczyk, Melisa Belanger, Zackary Cope, Kate E. Foley, Benjamin A. Logsdon, Lara M. Mangravite, Stacey J. Sukoff Rizzo, Paul R. Territo, Gregory W. Carter, Michael Sasner, Bruce T. Lamb, Gareth R. Howell

**Affiliations:** ^1^The Jackson Laboratory, Bar Harbor, ME, United States; ^2^Stark Neurosciences Research Institute, School of Medicine, Indiana University Bloomington, Indianapolis, IN, United States; ^3^Department of Medicine—Aging Institute, University of Pittsburgh School of Medicine, Pittsburgh, PA, United States; ^4^Sage Bionetworks, Seattle, WA, United States

**Keywords:** ApoE4, TREM2, mouse model, MODEL-AD, late-onset AD

## Abstract

Late-onset Alzheimer’s disease (AD; LOAD) is the most common human neurodegenerative disease, however, the availability and efficacy of disease-modifying interventions is severely lacking. Despite exceptional efforts to understand disease progression *via* legacy amyloidogenic transgene mouse models, focus on disease translation with innovative mouse strains that better model the complexity of human AD is required to accelerate the development of future treatment modalities. LOAD within the human population is a polygenic and environmentally influenced disease with many risk factors acting in concert to produce disease processes parallel to those often muted by the early and aggressive aggregate formation in popular mouse strains. In addition to extracellular deposits of amyloid plaques and inclusions of the microtubule-associated protein tau, AD is also defined by synaptic/neuronal loss, vascular deficits, and neuroinflammation. These underlying processes need to be better defined, how the disease progresses with age, and compared to human-relevant outcomes. To create more translatable mouse models, MODEL-AD (Model Organism Development and Evaluation for Late-onset AD) groups are identifying and integrating disease-relevant, humanized gene sequences from public databases beginning with *APOEε4* and Trem2*R47H, two of the most powerful risk factors present in human LOAD populations. Mice expressing endogenous, humanized *APOEε4* and Trem2*R47H gene sequences were extensively aged and assayed using a multi-disciplined phenotyping approach associated with and relative to human AD pathology. Robust analytical pipelines measured behavioral, transcriptomic, metabolic, and neuropathological phenotypes in cross-sectional cohorts for progression of disease hallmarks at all life stages. *In vivo* PET/MRI neuroimaging revealed regional alterations in glycolytic metabolism and vascular perfusion. Transcriptional profiling by RNA-Seq of brain hemispheres identified sex and age as the main sources of variation between genotypes including age-specific enrichment of AD-related processes. Similarly, age was the strongest determinant of behavioral change. In the absence of mouse amyloid plaque formation, many of the hallmarks of AD were not observed in this strain. However, as a sensitized baseline model with many additional alleles and environmental modifications already appended, the dataset from this initial MODEL-AD strain serves an important role in establishing the individual effects and interaction between two strong genetic risk factors for LOAD in a mouse host.

## Introduction

Alzheimer’s disease (AD) is the most common form of dementia, currently affecting 5.8 million patients in the United States (Alzheimer’s Association, 2020), ~95% of which are late-onset (LOAD; Cacace et al., 2016). Unfortunately for patients and their families, interventions approved to delay or reverse AD-related neurodegeneration have yet to materialize (U.S. Department of Health and Human Services, 2021), despite promising new therapies aimed at decreasing amyloid burden (U.S. Food and Drug Administration, 2021). LOAD is a heterogeneous disease defined by a few widely accepted hallmarks: extracellular amyloid plaques, intracellular tau tangles, vascular dysfunction, immune activation, synapse loss, neuron death, and cognitive decline (Blennow et al., 2006). Dissecting the etiology of these properties in animal models has provided key insights into our understanding of the disease and strategies to treat AD, however, human clinical trials of resultant therapeutics have not yielded an approvable drug.

In 2015, the National Institutes on Aging (NIA) aimed to accelerate the AD drug testing pipeline by promoting the design of more predictive preclinical studies that better translate to human disease (U.S. Department of Health and Human Services, 2021). Current popular animal models of AD rely on the expression of transgenic alleles to promote the aggregation of amyloid plaques or neurofibrillary tangles as drivers of subsequent disease processes, notably during adolescence and early adulthood (Sasaguri et al., 2017). Therefore, these overexpression models do not fully capitulate the breadth of the disease observed in human patients, particularly elderly populations; many underlying biological pathways affected in human disease are not observed in these mice (Logsdon et al., 2019). From this need, the MODEL-AD (Model Organism Development and Evaluation for Late-onset AD) consortium was established to provide the research community with the next generation of AD animal models (Oblak et al., 2020). In an effort to design and validate novel mouse models of LOAD that better mimic human disease, we opted to create knock-in humanized coding and non-coding LOAD risk variants expressed at endogenous levels. To implement these considerations in the development of a new generation of preclinical animal models, candidate humanized gene variants or SNPs from a number of public data repositories (ADSP, AMP-AD, ADGC, ADNI, ROS/MAP, IGAP, M^2^OVE-AD, Resilience-AD, ACT) are being identified and engineered into existing mouse genes under endogenous promoters (Karch and Goate, 2015). As a heterogenous disease, it is unlikely that a single genetic alteration will promote the complex set of endophenotypes observed in humans. Rather, a combination of genetic and/or environmental risk factors is likely needed to phenocopy human disease and a better understanding of the effects of the complex interrelationships between risk factors is required to identify prospective therapeutic avenues. To this end, new mouse strains and conditions better replicating human LOAD are needed to improve the clinical translation of mouse therapies in human patients.

To date, approximately 40 loci have been identified through genetic and genome-wide association studies that increase the risk for AD (Dourlen et al., 2019; Andrews et al., 2020; Bellenguez et al., 2020). The strongest of these risk factors include the ε4 allele of apolipoprotein E (*APOE*) and point mutations in triggering receptor expressed on myeloid cells 2 (*TREM2)* locus (Allen et al., 2016; Cacace et al., 2016; Wang et al., 2018; Logsdon et al., 2019; Andrews et al., 2020; Bellenguez et al., 2020). These mutations were characterized in C57BL/6J mice alone and each correlated by gene-expression analysis to human transcripts to predict risk. Furthermore, by expressing both risk alleles in concert, genetic interaction and parallel molecular pathways could be investigated for dominance or synergistic effects that may promote human-like disease signatures.

*APOE*, the strongest genetic determinant of LOAD risk, has been the focus of extensive investigation for many years (Bu, 2009; Arnold et al., 2020; Foley et al., 2020), including the development of multiple mouse models expressing transgenes or targeted mutations (Xu et al., 1996; Raber et al., 1998; Sun et al., 1998; Knouff et al., 1999; Huber et al., 2000; Tesseur et al., 2000; Lesuisse et al., 2001; Bien-Ly et al., 2012). *APOE* is important in lipoprotein metabolism and immunoregulation strongly associated with cardiovascular and Alzheimer’s disease (Karch and Goate, 2015; Allen et al., 2016; Cacace et al., 2016; Yamazaki et al., 2016; Wang et al., 2018; Logsdon et al., 2019; Andrews et al., 2020; Bellenguez et al., 2020). Three isoforms of *APOE* are expressed in the human population: *APOE*ε2, *APOE*ε3, and *APOE*ε4 which confer increasing risk of LOAD, respectively. Compared to *APOE*ε3 carriers, the ε4 isoform of *APOE* increases AD risk and decreases the age of diagnoses (Saunders et al., 1993; Strittmatter et al., 1993). The three isoforms differ by one amino acid each at positions 112 and 158 that has profound effects on their functions: *APOE*ε2 (Cys112, Cys158); *APOE*ε3 (Cys112, Arg158); and *APOE*ε4 (Arg112, Arg158). The APOE protein has been shown to stimulate binding, transport, and metabolism of lipoproteins, major cholesterol transporters in the central nervous system (CNS) resulting in hypocholesteremia, tight junction failure, and vascular dysregulation (Maezawa et al., 2006c; Jeong et al., 2019). Additionally, reports of mice carrying human *APOEε4*-targeted replacement allele show evidence of blood-brain barrier (BBB) leakiness (Methia et al., 2001; Bell et al., 2012), immune alterations (Laskowitz et al., 2000; Maezawa et al., 2006a,b; Chung et al., 2016), synaptic disfunction (Safieh et al., 2019), and behavior deficits (Wang et al., 2005). Most importantly, APOE is also implicated in beta-amyloid and tau clearance (Verghese et al., 2013; Shi et al., 2017). However, many of the aspects of LOAD are not recapitulated upon expression of *APOE*ε4 alone, including the formation of beta-amyloid plaques and neurofibrillary tangles (Wang et al., 2005).

*TREM2* encodes a member of a receptor signaling complex with TYRO protein tyrosine kinase binding (TYROBP) protein, which activates microglia, macrophages, and dendritic cells during damage and immune responses (Ma et al., 2015; Keren-Shaul et al., 2017; Krasemann et al., 2017) functioning in processes like debris clearance (Kleinberger et al., 2017) and amyloid plaque response (Jay et al., 2015; Wang et al., 2015; Yuan et al., 2016). Single nucleotide polymorphisms (SNP) found in *TREM2* have been shown to regulate microglial function (Painter et al., 2015; Krasemann et al., 2017; Mazaheri et al., 2017), the most widely studied being the R47H missense mutation in exon 2. The TREM2*R47H mutation triples the carrier’s likelihood of Alzheimer’s disease (Allen et al., 2016; Cacace et al., 2016; Wang et al., 2018; Logsdon et al., 2019; Andrews et al., 2020; Bellenguez et al., 2020). The increased risk is suggested to be, in part, the result of decreases in the microglial receptor’s interactions with ligands (phospholipids, APOE, and beta-amyloid) yielding chronic dysfunction in microglial phagocytosis and inflammatory pathways (Karch and Goate, 2015; Ma et al., 2015; Painter et al., 2015).

Here we describe the application of a multi-faceted, cross-sectional phenotyping approach developed by MODEL-AD that included biometrics, behavioral assays, transcriptomics, neuroimaging, and immunohistochemistry to assess AD-relevant phenotypes in mice expressing combinations of humanized *APOE*ε4 and Trem2*R47H alleles.

## Materials and Methods

### Model Backgrounds

All animals were obtained from The Jackson Laboratory. Mouse models of Late-onset Alzheimer’s disease (LOAD) developed by MODEL-AD (Model Organism Development and Evaluation for Late-onset Alzheimer’s Disease) are congenic to the C57BL/6J (JAX# 000664; B6) strain. Genetic variants, identified from human data compiled by the AMP-AD (Accelerating Medicines Partnership-Alzheimer’s Disease) project, expressed in MODEL-AD-generated strains are listed on the MODEL-AD strain table at https://www.model-ad.org/strain-table/, along with relevant links for allele descriptions, data, distribution, and legal disclaimers.

### B6J.APOE4.Trem2*R47H (LOAD1) Mice

The B6.APOE4.Trem2*R47H (LOAD1) double mutant strain created at The Jackson Laboratory in Bar Harbor, Maine carries two primary risk alleles found in Alzheimer’s disease patients. The humanized ApoE knock-in allele, in which a portion of the mouse *Apoe* gene (exons 2, 3, a majority of exon 4, and some 3’ UTR sequence) of the mouse *Apoe* gene was replaced by the corresponding sequence of the human APOE4 gene (available as B6(SJL)-Apoe^tm1.1(APOE*4)Adiuj^/J^1^, Foley et al., 2020). The second allele, *Trem2*, contains the R47H point mutation and two additional silent mutations (available as C57BL/6J-Trem2^em1Adiuj^/J^2^). The human R47H variant, when expressed in mouse brains, also confers a novel splice variant due to a cryptic slice acceptor site in exon 2 (Xiang et al., 2018). See additional information in the Jackson Laboratory APOE4.Trem2R47H mouse (JAX strain #028709) strain data sheet. APOE4.Trem2R47H mouse strain data sheet at https://www.jax.org/strain/028709.

### Experimental Cohorts

To decipher how these two strong risk factors drive AD-relevant phenotypes, we created a double homozygous B6.APOE4.Trem2*R47H model, accompanied by single genotype controls, on a C57BL/6J (B6) background (Table 1). In appreciation of sexual dimorphism observed in human aging and disease, cohorts of males and females were established for phenotyping at 4-, 8-, 12- and 24-months using a cross-sectional design. To determine whether the LOAD risk variants *APOE*ε4, Trem2*R47H, or the combination produced *in vivo* phenotypes independent from normal healthy aging, a comprehensive cross-sectional phenotyping battery was conducted and included *in vivo* frailty assessments, metabolic screening, microbiome sampling, biomarker evaluation, behavioral phenotyping, and *in vivo* imaging. Postmortem brain tissue was further examined for transcriptomic and neuropathological indications of disease. All accumulated data sets and observations are disseminated for public availability (Kotredes, 2020).

**Table 1:** Description of novel mouse strains expressing human LOAD risk alleles.

Common name	JAX stock #	Strain	Background	Gene location	Allele name	Allele type	Additional considerations
**B6**	000664	C57BL/6J	-	-	-	-	-	
Trem2*R47H	027918	C57BL/6J- Trem2^em1 Adiuj/J^	C57BL/6J	Chr17:4834 6401- 48352276	Trem2 R47H KI	Cas9 endonuclease- mediated (humanized sequence)	- Two silent mutations (lysine AAG>AAA and alanine GCC>GCA) into *Trem2* - R47H mutation also introduces a cryptic splice acceptor site in exon 2, creating a novel splice variant with a deletion of 119bp at the 5’ end of exon 2.
**APOE4**	027894	B6(SJL)- *Apoe*^tm1.1(APOE*4) Adiuj/J^	C57BL/6J	Chr7:19696 109-19699188	APOE4 KI	FRT site flanked PGK- neo cassette targeted mutation (gene replacement)	- Exons 2, 3, and a majority of exon 4 of the mouse *Apoe* gene were replaced by exons 2, 3, and 4 of the human *APOE* gene sequence (including a portion of the 3’ UTR sequence) - Expression of FLP recombinase was used to remove the FRT site flanked PGK-neo cassette and subsequently backcrossed to remove FLP recombinase
APOE4. Trem2*R47H	028709	B6(SJL)- *Apoe*^tm1.1(APOE*4)Adiuj^ Trem2^em1Adiuj/J^	C57BL/6J	(see above)	(see above)	(see above)	(see above)

Mouse strains expressing Trem2*R47H and *APOEε4* human late-onset Alzheimer Disease risk factors, alone or in combination, developed on the C57BL/6J background and distributed by The Jackson Laboratory (https://www.jax.org/mouse-search).

### Animal Housing Conditions

All experiments were approved by the Animal Care and Use Committee at The Jackson Laboratory and the Institutional Animal Care and Use Committee at Indiana University. To minimize gene expression variation between mice, animal housing conditions were replicated between both Bar Harbor and Indianapolis campuses. Mice were bred in the mouse facility at Indiana University or The Jackson Laboratory and maintained in a 12/12-h light/dark cycle, consisting of 12 h-ON 7 am-7 pm, followed by 12 h-OFF. Room temperatures are maintained at 18–24°C (65–75°F) with 40–60% humidity. All mice were housed in positive, individually ventilated cages (PIV). Standard autoclaved 6% fat diet (Purina Lab Diet 5K52) was available to the mice *ad-lib*, as was water with acidity regulated from pH 2.5–3.0. All breeder and experimental mice were housed in the same mouse room and were aged together. All behavioral characterization was conducted in the Mouse Neurobehavioral Core Facility (MNBF) at The Jackson Laboratory. Briefly, mice were relocated from the housing room in which they were reared to the MNBF in an adjacent building on the Bar Harbor campus. Mice were individually housed at minimum 5 days prior to behavioral testing. The dedicated MNBF housing room consists of PIV caging with temperature controlled at a setting of 22 ± 1°C (72 ± 2°F) and hsumidity at 50 ± 20%. The testing facility was on a 12:12 L:D schedule (lights on at 6:00 am) with all testing performed during the light cycle (typically between 7:00 am and 5:00 pm, with the exception of wheel running which was continuous 24-h testing for up to 5 days). All subjects were randomized and counterbalanced for testing order across multiples of instrumentation and time of day for each test day, with a simplified testing ID number (e.g., #1-100), with all technicians blinded to genotype (e.g., coded as A, B, C, etc.). The blind was maintained throughout testing and until after the data were analyzed with no subjects or data excluded based on any mathematical outliers.

### Behavioral Testing

Behavioral tests were conducted as previously reported (Sukoff Rizzo et al., 2018) in the following order with at minimum a 1–2-day rest period between tests: Frailty assessment with core body temperature recording, open field test, spontaneous alternation, rotarod, and wheel-running activity. On each test day, subjects were transported from the adjacent housing room into the procedure room, tails were labeled with a non-toxic permanent marker with the assigned subject ID number, and subjects were left to acclimate undisturbed to the testing environment for a minimum 60 min prior to testing. Between subjects, all testing arenas were sanitized with 70% ethanol solution and dried prior to introducing the next subject. Lighting in the testing rooms were consistent with the housing room (~500 lux) unless where specifically noted. At minimum 5 days post the conclusion of behavioral testing, mice were sent for tissue harvesting.

### Frailty Assessment

Similar to as previously described (53), subjects were individually evaluated for the absence or presence of 26 aging-related characteristic traits and scored a 0, 0.5, or 1 (based on presence/absence, and severity) for each assessment by a trained observer, blind to genotype/age, and included the following assessments: alopecia; loss of fur color; dermatitis/skin lesions; loss of whiskers; coat condition; piloerection; cataracts; eye discharge/swelling; microphthalmia; nasal discharge; rectal prolapse; vaginal/uterine/penile; diarrhea; vestibular disturbance; vision loss assessed by visual placing upon the subject being lowered to a grid; menace reflex; tail stiffening; impaired gait during free walking; tremor; tumors; distended abdomen; kyphosis; body condition; breathing rate/depth; malocclusions; righting reflex. The frailty index score was calculated as the cumulative score of all measures with a maximum score of 26.

### Core Body Temperature

Core body temperature was recorded just prior to the conclusion of the frailty assessment *via* a glycerol lubricated thermistor rectal probe (Braintree Scientific product# RET 3; measuring 3/4" L 0.028 dia. 0.065 tip) inserted ~2 cm into the rectum of a manually restrained mouse for approximately 10 s. The temperature was recorded to the nearest 0.1°C (Braintree Scientific product#TH5 Thermalert digital thermometer).

### Open Field Activity

Versamax Open Field Arenas (40 cm × 40 cm × 40 cm; Omnitech Electronics, OH, USA) were used for this test. Arenas were housed within sound-attenuated chambers with lighting in the testing room and arenas consistent with the housing room (~500 lux). Mice were placed individually into the center of the arena and infrared beams recorded distance traveled (cm), vertical activity, and perimeter/center time. Data were collected in 5-min time-bins for a duration of 60 min.

### Spontaneous Alternation

Mice were acclimated to the testing room under ambient lighting conditions (~50 lux). A clear polycarbonate y-maze (in-house fabricated; arm dimensions 33.65 cm length, 6 cm width, 15 cm height) placed on top of an infrared reflecting background (Noldus, The Netherlands), surrounded by a black floor-to- ceiling curtain to minimize extramaze visual cues was used for this test. Mice were placed midway of the start arm (A), facing the center of the y for an 8-min test period and the sequence of entries into each arm is recorded *via* a ceiling-mounted infrared camera integrated with behavioral tracking software (Noldus Ethovision XT). Percent spontaneous alternation is calculated as the number of triads (entries into each of the three different arms of the maze in a sequence of three without returning to a previously visited arm) relative to the number of alteration opportunities.

### Rotarod Test for Motor Coordination

An accelerating Rotarod (Ugo-Basile; model 47600) is used for this test. Lighting in the testing room is consistent with the housing room (~500 lux). The trial began with mice being placed on the rotating rod (4 rpm), which accelerates up to 40 rpm over the course of 300 s. Each mouse is subjected to three consecutive trials with an ~1 min inter-trial interval to allow cleaning of the rod between trials. Latency to fall (sec) is measured. Subjects that fall upon initial placement on the rod, before acceleration begins, are scored as 0 s for that trial.

### Wheel Running Activity

Subjects were individually housed in a clean cage with a running wheel (Med-Associates, Vermont, USA) and with food and water ad libitum. The light cycle was identical to the housing room with 12:12 L:D (lights on at 6:00 am). Running wheels were equipped with a wireless transponder that recorded activity on the running wheels (revolutions) in sync with a computer that time stamps events. Mice were left undisturbed throughout the testing period with the exception of daily welfare checks. Data were evaluated for time spent running (min), total distance traveled (meters), and speed (revolutions per min) over the course of three 24-h periods.

### Behavioral Data Analysis

Prior to data analysis and while still blinded, results were adjusted to exclude data only from mice which could not be tested or which data was not available inclusive of any equipment failures, escape episodes, etc. Subjects were not excluded by any mathematical determination. Data were analyzed under coded genotypes (A, B, C, etc.) within sex, as one-way or two-way ANOVA as appropriate vs. sex- and age- matched WT control. The blind was revealed at the conclusion of the data analysis for interpretation.

### Fasting Blood Glucose Collection and Measurement

Fasted mice were placed into a fresh cage, free of food but with fresh water, at 6 am—the beginning of the light-ON cycle. Mice were made to fast for 6 h, until 12 pm, at which time blood glucose levels were analyzed. Prior to mouse restraint, a Contour Next EZ blood glucose monitor (Ascensia, Parsippany, NJ) was calibrated with Contour glucose control solution and Contour Next test strips. While restraining the animal, with a 5.0 mm lancet a stab incision was made into and perpendicular to the cheek, located dorsal to the cheek skin gland at a distance equal to the height of the eye and caudal distance equal to the length of the eye. One drop of blood, approximately 10 μl, was applied to a blood glucose test strip and readings were recorded.

### Fecal Collection

Parallel with the measurement of animal weight, animals were placed in a clean container on a scale. Mouse weight was recorded and upon production, a fecal sample was collected with forceps to prevent contamination. The sample was placed in a pre-marked 1.5 ml tube and snap-frozen immediately on dry ice. Container and forceps were cleaned with 70% ethanol before collecting from subsequent mice. Fecal samples were stored long term at −80°C until analyzed.

### Animal Anesthesia

Upon arrival at the terminal endpoint for each aged mouse cohort, individual animals were weighed prior to intraperitoneal administration of either: (A) ketamine (100 mg/kg) and xylazine (10 mg/kg); or (B) tribromoethanol (1 mg/kg). Routine confirmation of deep anesthesia was performed every 5 min by toe pinch. First confirming deep anesthetization *via* toe pinch, an incision along the ventral midline to expose the thorax and abdomen, followed by removal of the lateral borders of the diaphragm and ribcage revealed the heart. If desired, prior to perfusion blood and CSF samples must be collected. To perfuse the animal, a small cut was placed in the right atrium to relieve pressure from the vascular system before perfusing the animal transcardially with 1× PBS *via* injection into the left ventricle. Completion of perfusion and clearance of the vascular system was indicated by blanching of the liver.

### Whole Animal Perfusion

First confirming deep anesthetization *via* toe pinch, animals are secured to a surgical board or tray using needles or pins and abdomen wetted with 70% ethanol followed by an incision along the ventral midline along the entire ventral surface, exposing the underlying muscle of the thorax and abdomen. An additional incision is made into this underlying muscle and cut to puncture the diaphragm, taking care not to cut any major blood vessels or the lungs. To expose the heart the ribcage can be cut along the lateral borders and removed. A small incision is made in the right atrium of the heart to relieve diastolic pressure and begin the removal of blood from the vascular system. To clear the vascular system of all blood a butterfly catheter needle is inserted into the left ventricle attached to a perfusion pump. Approximately 10 ml of 1× PBS solution will clear the system of a 20 g animal. Once the system has been cleared of blood the liver will appear very pale and PBS will be noticed exiting the right atrium. At this time organs of interest were collected as indicated.

### Non-fasting Blood Collection and Analysis

Blood was collected by cardiac puncture from non-fasted, anesthetized animals (see Perfusion method) at harvest prior to incision of the right atrium and subsequent perfusion. A 25-gauge EDTA-coated needle, attached to a 1 ml syringe, is inserted into the right atrium of the exposed heart and the plunger gently pulled to slowly aspirate approximately 500 ml of blood, avoiding entrapping air in the syringe to prevent hemolysis. After removal of the needle from the syringe, the blood was slowly injected into a 1.5 ml EDTA coated MAP-K2 blood microtainer (363706, BD, San Jose, CA) on ice. Blood tubes were spun at 4°C and 4,388× *g* for 15 min. Blood serum is then removed and aliquoted equally into three replicate 1.5 ml tubes on ice. Tubes were then snap-frozen on dry ice and stored long-term at −80°C. Thawed blood plasma collected from non-fasted mice was then analyzed by Beckman Coulter AU680 chemistry analyzer (Beckman Coulter, Brea, CA) and Siemens Advia 120 (Germany) for levels of non-fasted glucose, total cholesterol, LDL (low-density lipoproteins), HDL (high-density lipoproteins), triglycerides, and NEFA (non-essential fatty acids).

### Brain Harvest

Anesthetized and subsequently perfused animals were decapitated, and heads submerged quickly in cold 1× PBS. The brain was carefully removed from the skull, weighed, and divided midsagitally, into left and right hemispheres, using a brain matrix. The right hemisphere was quickly homogenized on ice and equally aliquoted into three cryotubes for metabolomic, proteomic, and transcriptomic analysis. Cryotubes were immediately snap-frozen on dry ice and stored long-term at −80°C. The left hemisphere was immediately placed in 5 ml 4% PFA at 4°C for no less than 24 h, but no longer than 30 h. The left hemisphere was then moved from PFA solution to 10 ml 15% sucrose at 4°C for 24 h, or until it sinks in the sucrose, when it was then transferred to a 30% sucrose for 24 h at 4°C, or until it sinks in the solution. The left hemisphere was then removed from 30% sucrose solution, snap-frozen on a flat mold, cut-side down, floating in 2-methyl butane solution cooled by dry ice. Once frozen the left hemisphere is then placed into a cryotube and stored at −80°C until used for microtome sectioning and immunohistochemistry analysis.

### Immunohistochemistry and Microscopy Imaging

During harvest, whole mouse brains were removed and weighed. Using a brain matrix, left and right hemispheres were separated along the midsagittal plane. The left hemisphere was placed in 5 ml of 4% PFA at 4°C overnight, then moved to 10 ml of 15% sucrose at 4°C overnight, before finally being incubated in 10 ml of 30% sucrose at 4°C overnight or until the brain sinks to the bottom of the tube. The left hemisphere was then snap-frozen and stored at −80°C until sectioned. Left hemispheres (see preparation in Brain harvest method) were cut *via* Thermo Scientific HM430 sliding microtome at 25 μm thickness. Coronal brain tissue sections were oriented to capture the cortex and hippocampus at approximately Bregma: −2.75 mm and Interaural 1.05 mm. Each section was placed into cryoprotectant buffer (37.5% 1× PBS, 31.25% glycerol, 31.25% ethylene glycol) for immediate use or long-term storage at −20°C. Each section was tracked so as to store 100 sections equally distributed over 10 groups, so each of the 10 groups had equal representation of the 10 sections from the forebrain to the hindbrain and the hippocampus. Of these 10 groups of 10 25-micron sections, seven will be used for standardized staining combinations highlighting cell types and markers of interest: (1) Vascular [CD31/Iba1/Fibrin/DAPI]; (2) Neuritic plaques [Lamp1/Iba1/X34]; (3) Astrocytes and microglia [GFAP/Iba1/S100b/DAPI]; (4) Neurons [NeuN/Ctip2/DAPI]; (5) Plaques [ThioS]; (6) Luxol Fast Blue/Cresyl Violet; (7) Haematoxylin and eosin; and (8) Prussian blue (Iron stain). Floating sections were then blocked prior to immunohistochemical staining and mounting. After blocking slides with 10% normal donkey serum or normal goat serum diluted in 1× PBS+0.5%Triton wash buffer all antibodies were washed floating in 1× PBT (1× PBS with 0.5% Triton) wash buffer after blocking for 1 h at room temperature on a shaker in 10% NGS (normal goat serum) or 10% NDS (normal donkey serum) in 1× PBT. Secondary antibodies were incubated in 10% NGS or NDS in 1× PBT for 1 h at room temperature, followed by washes in 1× PBT before mounting onto the slides. Each staining combination of 10 sections was placed onto one slide. DAPI, X34, and ThioS stains were performed on the slide following immunostaining. Slides were then imaged on a Leica Versa slide scanner, automated fluorescent microscope system (Leica, Allendale, NJ). For further analysis, regions of the cortex and hippocampus were processed using CellProfiler (Cambridge, MA) or Imaris (Bitplane, Concord, MA) software to quantify cell counts, fluorescence intensity, and surface area ratios. [CD31 (R&D Systems, MAB3628, 1:500), Iba1 (Wako, 019-19741, 1:300), Fibrin (abcam, ab118533, 1:500), DAPI (1:1,000), Lamp1 (abcam, ab25245, 1:500), X34 (0.04% in 40% ethanol), GFAP (Origene, AP31806PU-N, 1:1,000), S100b (Thermo Fisher, PA175395, 1:1,000), NeuN (abcam, ab104225, 1:500), Ctip2 (abcam, ab18465, 1:1,000), and ThioS (1% in 50% ethanol)].

### *In vivo* Imaging Radiopharmaceuticals and Study Population

Regional brain glycolytic metabolism was monitored using 2-[18F]-fluoro-2-deoxy-D-glucose (18F-FDG) and was synthesized, purified, and prepared according to established methods (Yu et al., 2006), where clinical unit doses ranging from 185–370 MBq (5–10 mCi) were purchased from PETNet Indiana (PETNET Solutions Inc). To evaluate region brain perfusion, Copper(II) pyruvaldehyde bis(N4-methylthiosemicarbazone) labeled with 64Cu (64Cu-PTSM) was synthesized, purified, and unit doses (i.e., 370–740 MBq (10–25 mCi)) dispensed by the PET Radiochemistry Core Facility at Indiana University according to methods described previously (Green, 1987; Mathias et al., 1990).

### Magnetic Resonance Imaging (MRI)

To provide high contrast gray matter images, at least 2 days prior to PET imaging, mice were induced with 5% isoflurane (balance medical oxygen), placed on the head coil, and anesthesia maintained with 1–3% isoflurane for scan duration. High-resolution T2-weighted (T2W) MRI images were acquired using a 3T Siemens Prisma clinical MRI scanner outfitted with a dedicated 4 channel mouse head coil and bed system (RapidMR, Columbus OH). Images were acquired using a SPACE3D sequence (Algin and Ozmen, 2012) using the following acquisition parameters: TA: 5.5 min; TR: 2,080 ms; TE: 162 ms; ETL: 57; FS: On; Ave: 2; Excitation Flip Angle: 150; Norm Filter: On; Restore Magnetization: On; Slice Thickness 0.2 mm: Matrix: 171 × 192; FOV: 35 × 35 mm, yielding 0.18 × 0.18 × 0.2 mm resolution images. At the completion of the imaging period, mice we returned to their warmed home cages and were allowed to recover.

### Positron Emission Tomography (PET) Imaging

To evaluate changes in cerebral glycolysis (18F-FDG) and cerebral perfusion (64Cu-PTSM) mice were placed in a restrainer and consciously injected into the peritoneal or tail vein, respectively, with 3.7–11.1 MBq (0.1–0.3 mCi) of purified, sterile radiotracer, where the final volume did not exceed 10% of the animal’s body weight. Each animal was returned to its warmed home cage and allowed 30 min (18F-FDG) or 5 min (64Cu-PTSM) to allow for uptake and cellular trapping (Sokoloff, 1977; Mathias et al., 1991a). Post-uptake, mice were induced with 5% isoflurane gas, placed on the scanner imaging bed, and anesthesia maintained at 1–3% isoflurane (balance medical oxygen) during acquisition. In all cases, calibrated PET acquisition was performed in list mode for 15 (18F-FDG) or 30 (64Cu-PTSM) min on an IndyPET3 scanner (Frese et al., 2003), where random prompts did not exceed 10% of the total prompt rate. Post-acquisition, the images were reconstructed into a single-static image with a minimum field of view of 60 mm using filtered-back-projection (FBP), and were corrected for decay, random coincidence events, and dead-time loss (Soon et al., 2007).

### *In vivo* PET/CT Imaging

To assess regional glycolysis and tissue perfusion, mice will be non-invasively imaged *via* PET/CT (*n* = 10 mice/sex/genotype/age). To measure regional blood flow, copper-pyruvaldehyde-bis (N4-methylthiosemicarbazone; 64Cu-PTSM; Green, 1987), which has a very high first pass (>75%) extraction (Mathias et al., 1991b), and glutathione reductase redox trapping of copper (Mathias et al., 1991b), will be administered *via* tail vein in awake subjects and will be given a 2 min uptake period prior to imaging. To measure regional glycolytic metabolism, 2-fluoro-2-deoxyglucose (18F-FDG) will be administered *via* tail vein in awake subjects and mice will be given a 30 min uptake period prior to imaging. Post uptake, mice will be induced with 5% isoflurane (95% medical oxygen) and maintained during acquisition with 1–2% isoflurane at 37°C. To provide both anatomical structure and function, PET/CT imaging will be performed with a Molecubes β-X-CUBE system (Molecubes NV, Gent Belgium). For PET determination of blood flow and metabolism, calibrated listmode PET images will be acquired on the β-CUBE and reconstructed into a single-static image using ordered subset expectation maximization (OSEM) with 30 iterations and three subsets (Krishnamoorthy et al., 2018). To provide anatomical reference, and attenuation maps necessary to obtain fully corrected quantitative PET images, helical CT images were acquired with tube voltage of 50 kV, 100 mA, 100 μm slice thickness, 75 ms exposure, and 100 μm resolution. In all cases, images will be corrected for radionuclide decay, tissue attenuation, detector dead-time loss, and photon scatter according to the manufacturer’s methods (Krishnamoorthy et al., 2018). Post-acquisition, all PET and CT images will be co-registered using a mutual information-based normalized entropy algorithm (Studholme et al., 1998) with 12 degrees of freedom and mapped to stereotactic mouse brain coordinates (Paxinos and Franklin, 2012). Finally, to quantify regional changes, voxels of interest (VOI) for 27 brain (54 bilateral) regions will be extracted and analyzed for SUVR according to published methods (Dandekar et al., 2007).

### Autoradiography

To provide secondary confirmation of the *in vivo* PET images, and to quantify tracer uptake regionally, brains were extracted post rapid decapitation, gross sectioned along the midline, slowly frozen on dry ice, then embedded in cryomolds with Optimal Cutting Temperature (OCT) compound (Tissue-Tek). Thin frozen sections (20 um) were obtained *via* cryotomy at prescribed bregma targets (*n* = 6 bregma/mouse, 6 replicates/bregma) according to stereotactic mouse brain coordinates (Franklin and Paxinos, 2013). Sections were mounted on glass slides, air dried, and exposed on BAS Storage Phosphor Screens (SR 2040 E, Cytiva Inc.) for up to 12 h. Post-exposure, screens were imaged *via* Typhoon FL 7000IP (GE Medical Systems) phosphor-imager at 25 um resolution along with custom 18F or 64Cu standards described previously (Territo et al., 2017).

### Image Analysis

All PET and MRI images were co-registered using a ridged-body mutual information-based normalized entropy algorithm (Studholme et al., 1997) with 9 degrees of freedom, and mapped to stereotactic mouse brain coordinates (Franklin and Paxinos, 2013) using Analyze 12 (AnalyzeDirect, Stilwell KS). Post-registration, 56 regions bilateral regions were extracted *via* brain atlas and averaged to yield 27 unique volumes of interest that map to key cognitive and motor centers that include: Agranular Insular Cortex; Auditory Cortex; Caudate Putamen, Cerebellum; Cingulate Cortex; Corpus Callosum; Dorsolateral Orbital Cortex; Dorsintermed Entorhinal Cortex; Dysgranular Insular Cortex; Ectorhinal Cortex; Fornix; Frontal Association Cortex; Hippocampus; Lateral Orbital Cortex; Medial Orbital Cortex; Parietal Cortex; Parietal Association Cortex; Perirhinal Cortex; Prelimbic Cortex; Primary Motor Cortex; Primary Somatosensory Cortex; Retrosplenial Dysgranular Cortex; Secondary Motor Cortex; Secondary Somatosensory Cortex; Temporal Association Cortex, Thalamus; Ventral Orbital Cortex; Visual Cortex. For autoradiographic analysis, tracer uptake was quantified on hemi-coronal sections by manually drawing regions of interest for 17 regions of interest (i.e., Auditory Cortex, Caudate Putamen, Cerebellum, Cingulate Cortex, Corpus Callosum, Dorso-intermed Entorhinal Cortex, Dysgranular Insular Cortex, Ectorhinal Cortex, Hippocampus, Hypothalamus, Medial Septum, Primary Motor Cortex, Primary Somatosensory Cortex, Retrosplenial Dysgranular Cortex, Temporal Association Cortex, Thalamus, Visual Cortex) on calibrated phosphor screen at bregma 0.38, −1.94, and −3.8 mm using MCID (InterFocus Ltd). To permit dose and brain uptake normalization, Standardized Uptake Value Ratios (SUVR) relative to the cerebellum were computed for PET and autoradiograms for each subject, genotype, and age as follows:


$$SUVR(s,R,g,a)=\frac{R(s,g,a)}{R(s,g,a)}$$

where, *s*, *g*, *a*, *R*, and *C* are the subject, genotype, age, region/volume of interest, cerebellum region/volume of interest. In all cases, region/volumes of interest were analyzed for differences with time and genotype using a Two-Way ANOVA (Prism, GraphPad Inc.), where significance was taken at *p* < 0.05.

### Immunoprecipitation

Tissue samples were homogenized in tissue protein extraction reagent (T-PER ThermoScientific) supplemented with protease and phosphatase inhibitors cocktail (Sigma-Aldrich). Protein concentration was measured using bicinchoninic acid (BCA; Pierce). Immunoprecipitation was performed by incubating a total of 1,500 μg of brain protein extract with 1 μg of biotinylated sheep anti-Trem2 antibody (RnD systems BAF1729) overnight at 4°C, followed by incubation with streptavidin sepharose beads (CST 3419) for 6 h at 4°C, washed three times with ice cold PBS with 0.1% Tween 20. Protein was eluted in sample loading buffer with 1 mM DTT followed by separation *via* Western blot (see below).

### Western Immunoblot

Snap-frozen right hemispheres were homogenized by hard tissue homogenizer (USA Scientific, Ocala, FL) and lysed in 1 ml RIPA buffer (R0278, Sigma, St. Louis, MO) supplemented with protease and phosphatase inhibitor reagents (1861281, Thermo Fisher Scientific, Waltham, MA). Lysates were incubated for 1 h at 4°C before pelleting insoluble proteins by spinning at 4°C, 11,000× *g* for 15 min. Protein concentration was determined by Bradford protein assay (Biorad, Hercules, CA), according to the manufacturer’s instructions. Samples were mixed with 10× Laemlli buffer (42556.01, Amsbio, Cambridge, MA), boiled for 10 min, and run on 12% SDS PAGE gels (456-1044, BioRad) with a colorimetric ladder (RPN800E, GE, Boston, MA). Gels were transferred to PVDF membranes for immunoblotting and imaging using an iBlot2 dry blotting system (Thermo Fisher). Membranes were blocked in 5% non-fat dry milk in 1× PBS+0.1% Tween20 for 1 h prior to incubating with primary antibodies diluted in 5% non-fat dry milk in 1× PBS+0.1% Tween20 for 1 h at room temperature. Membranes were washed in 1× PBS+0.1% Tween20 before incubating with secondary antibodies diluted in 5% non-fat dry milk in 1× PBS+0.1% Tween20. HRP-conjugated secondary antibodies targeting primary antibody host IgG were incubated at 1 h at room temperature. Membranes were washed in 1× PBS+0.1% Tween20 before digital imaging with SuperSignal West Pico PLUS chemiluminescent substrate (34579, Thermo Fisher). Images for immunoblot were quantified using ImageJ 1.8.0 version. Proteins of interest were visualized with the following primary antibodies against: ACTIN (Abcam, ab179467), GAPDH (abcam, ab9483), APOE4 (Novus, NBP1-49529), TREM2 (R&D Systems, MAB1729), and Alpha-tubulin (Sigma-Aldrich, T9026-100UL).

### Cytokine Panel Assay

Hemibrains were homogenized in tissue homogenization buffer containing fresh protease inhibitor cocktail and aliquoted. The supernatant was utilized for the cytokine analysis. Mouse hemibrain samples were assayed in duplicate using the MSD mouse proinflammatory Panel I, a highly sensitive multiplex enzyme-linked immunosorbent assay (ELISA). The panel quantifies 10 cytokines: interferon gamma (IFN-γ), interleukin (IL)-1β, IL-2, IL-4, IL-6, IL-8, IL-10, IL-12p70, IL-13, and tumor necrosis factor α (TNFα) from a single small sample volume (25 μl) using an electrochemiluminescent detection method (MesoScale Discovery, Gaithersburg, MD, USA). The mean intra-assay coefficient for each cytokine was <8.5%, based on cytokine standards. Any value that was below the lowest limit of detection (LLOD) for the cytokine assay was replaced with $\frac{1}{2}$ LLOD of the assay for statistical analysis.

### RNA-Sequencing Experimental Design

RNA-Seq data were obtained from whole left hemisphere brain samples from APOE4 KI mouse, carrying a humanized version of the prominent *APOEε4* genetic risk factor for LOAD, and the Trem2*R47H mouse, carrying a rare deleterious variant R47H allele of *Trem2* gene. In addition, a mouse model expressing both human *APOEε4* and the Trem2*R47H mutation was used to compare the transcriptional changes in mice carrying both variants to mice carrying only a single risk allele and B6 controls. Whole-brain left hemispheres were collected at 4, 8, 12, and 24 months of age from both sexes.

### RNA Sample Extraction

Total RNA was extracted from snap-frozen right brain hemispheres using Trizol (Invitrogen, Carlsbad, CA). mRNA was purified from total RNA using biotin-tagged poly dT oligonucleotides and streptavidin-coated magnetic beads and quality was assessed using an Agilent Technologies 2100 Bioanalyzer (Agilent, Santa Clara, CA).

### RNA-Sequencing Assay Library Preparation

Sequencing libraries were constructed using TruSeq DNA V2 (Illumina, San Diego, CA) sample prep kits and quantified using qPCR (Kapa Biosystems, Wilmington, MA). The mRNA was fragmented, and double-stranded cDNA was generated by random priming. The ends of the fragmented DNA were converted into phosphorylated blunt ends. An “A” base was added to the 3’ ends. Illumina^®^-specific adaptors were ligated to the DNA fragments. Using magnetic bead technology, the ligated fragments were size-selected and then a final PCR was performed to enrich the adapter-modified DNA fragments since only the DNA fragments with adaptors at both ends will amplify.

### RNA-Sequencing

Libraries were pooled and sequenced by the Genome Technologies core facility at The Jackson Laboratory. All samples were sequenced on Illumina HiSeq 4000 using HiSeq 3000/4000 SBS Kit reagents (Illumina), targeting 30 million read pairs per sample. Samples were split across multiple lanes when being run on the Illumina HiSeq, once the data was received the samples were concatenated to have a single file for paired-end analysis.

### RNA-Sequencing Data Processing

Sequence quality of reads was assessed using FastQC (v0.11.3, Babraham). Low-quality bases were trimmed from sequencing reads using Trimmomatic (v0.33; Bolger et al., 2014). After trimming, reads of length longer than 36 bases were retained. The average quality score was greater than 30 at each base position and sequencing depth was in range of 60–80 million reads. RNA-Seq sequencing reads from all samples were mapped to the mouse genome (version GRCm38.p6) using ultrafast RNA-Seq aligner STAR (v2.5.3; Dobin et al., 2013). To measure human *APOE* gene expression, we created a chimeric mouse genome by concatenating the human *APOE* gene sequence (human chromosome 19:44905754-44909393) into the mouse genome (GRCm38.p6) as a separate chromosome (referred to as chromosome 21 in chimeric mouse genome). Subsequently, we added gene annotation of the human *APOE* gene into the mouse gene annotation file. Additionally, we have also introduced annotation for novel *Trem2* isoform in mouse gene annotation file (GTF file), that is identical to primary transcript but truncated exon2 by 119 bp from its start position. Afterward, a STAR index was built for this chimeric mouse genome sequence for alignment, then STAR aligner output coordinate-sorted BAM files for each sample mapped to the chimeric mouse genome using this index. Gene expression was quantified in two ways, to enable multiple analytical methods: transcripts per million (TPM) using RSEM (v1.2.31; Li and Dewey, 2011), and raw read counts using HTSeq-count (v0.8.0; Anders et al., 2015).

### Differential Expression Analysis

Differential expression in mouse models was assessed using the R Bioconductor package DESeq2 (v1.16.1; Love et al., 2014). DESeq2 takes raw read counts obtained from HTSeq-count as input. Genes with the Benjamini-Hochberg corrected p-values < 0.05 were considered as significantly differentially expressed genes.

### Principal Component Analysis

We analyzed a total of 234 RNA-Seq samples originating from different mouse models of different ages and sex. First, the dispersion parameter for each gene was estimated using DESeq2 R package (Love et al., 2014). Afterward, we applied the variance stabilizing transformation (vst) function of DESeq2 (Love et al., 2014) to the read count data in order to produce a data matrix in which expression levels are homoscedastic. Finally, we extracted the principal components using the plot PCA function of DESeq2 in R.

### Functional Enrichment Analysis

Functional annotations and enrichment analyses were performed using the R Bioconductor package clusterProfiler (Yu et al., 2012), with Gene Ontology terms and KEGG pathways enrichment analyses performed using functions enrichGO and enrichKEGG, respectively. The function compareCluster was used to compare enriched functional categories of each gene module. The significance threshold for all enrichment analyses was set to 0.05 using Benjamini-Hochberg adjusted p-values.

### Human Post-mortem Brain Cohorts and Co-expression Module Identification

Whole-transcriptome data for human post-mortem brain tissue was obtained from the Accelerating Medicines Partnership for Alzheimer Disease-(AMP-AD) consortium, which is a multi-cohort effort to harmonize genomics data from human LOAD patients. Harmonized co-expression modules from the AMP-AD data sets were obtained from the AD Knowledge Portal (DOI: 10.7303/syn11932957.1). The human co-expression modules derive from three independent LOAD cohorts, including 700 samples from the ROS/MAP cohort, 300 samples from the Mount Sinai Brain bank, and 270 samples from the Mayo cohort. A detailed description of post-mortem brain sample collection, tissue and RNA preparation, sequencing, and sample QC has been provided elsewhere (Allen et al., 2016; De Jager et al., 2018; Wang et al., 2018). As part of a transcriptome-wide meta-analysis to decipher the molecular architecture of LOAD, 30 co-expression modules from seven different brain regions across the three cohorts have been recently identified (Logsdon et al., 2019). Briefly, Logsdon et al. (2019) identified 2,978 co-expression modules using multiple techniques across the different regions after adjusting for co-variables and accounting for batch effects (10.7303/syn10309369.1). A total of 660 co-expression modules were selected based on a specific enrichment in LOAD cases when compared to controls (10.7303/syn11914606). Finally, multiple co-expression module algorithms were used to identify a set of 30 aggregate modules that were replicated by the independent methods (Logsdon et al., 2019).

### Mouse-Human Correlation Analysis

First, we performed differential gene expression analysis for each mouse model compared to age and sex-matched B6 control mice using the limma (Ritchie et al., 2015) package in R. Afterward, we computed the correlation between changes in expression (log fold change) for all DE genes in a given module with the fold changes for each mouse model (specified by genotype, sex, diet, and age). Correlation coefficients were computed using cor.test function in R as:


(1) cor.test.(LogFC(h), LogFC(m)

where LogFC(h) is the log fold change in transcript expression of human AD patients compared to control patients and LogFC(m) is the log fold change in expression of mouse transcripts compared to control mouse models. LogFC values for human transcripts were obtained *via* the AD Knowledge Portal^3^.

### Statistical Analysis

Statistical analyses were constrained to comparisons between littermate-controlled subjects only (B6.APOE4 v.B6.APOE4.Trem2*R47H). The student’s t-test was employed on data sets differing by a single variable (e.g., age, sex, genotype). ANOVA was used in data sets where multiple factors are considered and combined for possible synergistic effects.

### Availability of Data and Materials

The LOAD1 data sets are available *via* the AD Knowledge Portal^4^. The AD Knowledge Portal is a platform for accessing data, analyses, and tools generated by the Accelerating Medicines Partnership (AMP-AD) Target Discovery Program and other National Institute on Aging (NIA)-supported programs to enable open-science practices and accelerate translational learning. The data, analyses, and tools are shared early in the research cycle without a publication embargo on a secondary use. Data is available for general research use according to the following requirements for data access and data attribution^5^.

For access to content described in this manuscript see: https://doi.org/10.7303/syn23631984.

## Results

### Biometric Profiles of APOE4.Trem2*R47H Mice Change With Age

**Figure 1:**
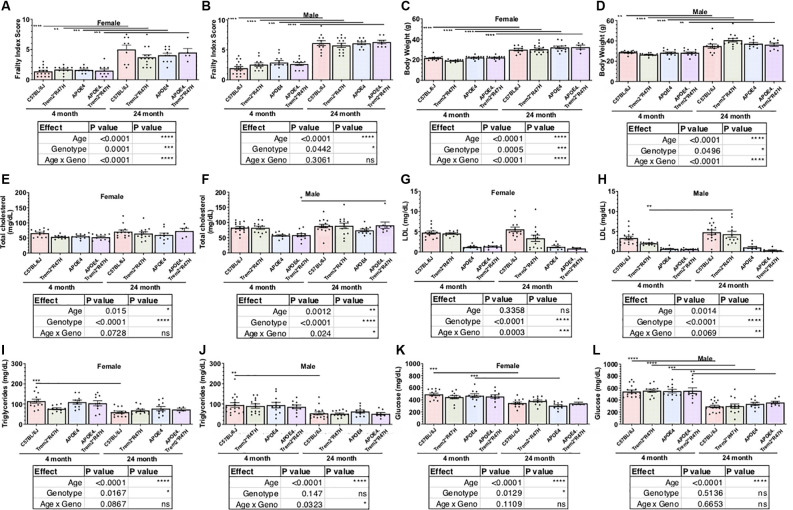
Age is primary factor driving increases in measures of frailty. Cross-sectional cohorts of young and old mice inspected for measures of physical well-being, or frailty **(A,B)**, including body weight **(C,D)**. Post-mortem, non-fasted, blood biochemistry analysis provided serum levels of cholesterol, lipoproteins, lipids, and glucose **(E–L)**. Age-dependent differences within genotype and sex determined by two-way ANOVA. Factor effects and effect interaction displayed in tables. *p < 0.05; **p < 0.01; ***p < 0.001. All alleles expressed were homozygous.

Comparison of biometric data from young (4 months) and aged mice (24 months), comprised of both sexes from four genotypes (Table 2), revealed the expected age-related accumulation in physical frailty characteristics (Figures 1A,B) with inverse correlations in body temperature (Supplementary Figure 1) and age-related increases in body weights (Figures 1C,D). However, effects due to genotype were not observed overall (see also Supplementary Table 1). Statistical analyses were constrained to contrasting only cohorts sharing littermates. Effects of age and genotype on variability were observed in datasets, as well as effective interaction, determined by ANOVA. These results confirm a strong aging effect in all assays. To determine the effects of *APOEε4* and Trem2*R47H on mouse metabolome, terminal non-fasted blood plasma levels of metabolites were measured. Homozygous expression of humanized *APOEε4* resulted in a significant decrease of non-fasted serum low-density lipoproteins (LDL) in the absence of corresponding decreases in total cholesterol (Figures 1E–H). Expected aging-specific-effects included decreases in glucose and triglyceride levels in both sexes independent of genotypes (Figures 1I–L). Other measurements and metabolic analytes included in our panel were unchanged between sex, age, and genotype cohorts (Supplementary Figures 1C–F and Supplementary Table 2). Additional cohorts, investigating *APOEε4* allele alone compared with littermate B6 controls, were aged to 12 months. Consistent with initial aging-related phenotypes in the 4- and 24-month cohorts, there was an expected increase in cumulative frailty scores, reductions in core body temperature, and increase in body weight in both sexes with no genotype-driven differences observed (Supplementary Figures 1G–I).

**Table 2:** Frailty assay and behavioral assays study population.

Mouse models	4M		8M		12M		24M	
	Male	Female	Male	Female	Male	Female	Male	Female
**C57BL/6J**	16	16	18	18	23	21	12	9
**APOE4 KI**	12	12	12	12	11	10	11	11
TREM2* R47H	12	11	12	12	13	11	13	14
APOE4.TREM2* R47H	11	10	12	11	12	10	8	6

### Age Is the Strongest Determinant of Performance by APOE4.Trem2*R47H Mice in Behavioral Assays

**Figure 2:**
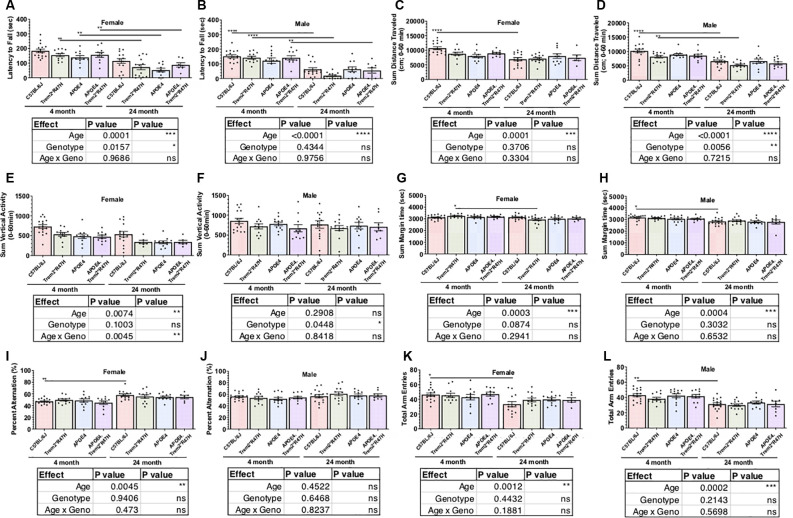
Behavioral testing of LOAD mice to identify functional neurophenotype. Performance of young and aged mice carrying combinations of APOE4 and Trem2*R47H alleles incorporated assays to measure neuromuscular coordination (rotarod; **A,B**), locomotor activity (open field; **C–F**), exploratory drive (open field; **G,H**), and spatial working memory (spontaneous alternation in Y-maze; **I–L**). Age-dependent differences within genotype determined by two-way ANOVA. Factor effects and effect interaction are displayed in tables. *p < 0.05; **p < 0.01; ****p < 0.001. All alleles expressed were homozygous.

As part of the comprehensive phenotypic characterization, all mice were evaluated through a behavioral testing pipeline consisting of open field, spontaneous alternation (y-maze), rotarod motor coordination, and running wheel activity assessments (Table 2; Supplementary Tables 2–5). Age-dependent impairments were observed across all genotypes from 4–24 months of age in locomotor activity as measured by open field and entries in the y-maze, motor coordination as measured by rotarod, and wheel- running activity (Figure 2; Supplementary Figures 2, 4). Spatial working memory was preserved up to 24 months of age with no deficits observed across genotype relative to 4 months of age. Interestingly, in the rotarod motor coordination assay, there was a greater impairment observed in B6.Trem2*R47H males relative to B6.APOE4.Trem2*R47H or B6.*APOE4* alone at 24 months (Figures 2A,B). Compared to 4-month-old animals, we observed a decrease in activity during the active period (dark phase) by 24 months (Supplementary Figure 4). At 24 months of age, home cage running wheel activity suggested a correlation between increased activity during the active period and expression of the *APOEε4* allele (both B6.*APOE4* and B6J.APOE4/Trem2*R47H mice) by way of total distance traveled and day-time activity in B6.Trem2*R47H mice (Supplementary Figures 4E,F). In addition to monitoring the physical wellness and behavior of otherwise healthy mice, we also wanted to track the effects of sex and genotype on animal longevity, in the absence of amyloid-associated AD. For this analysis, mortality was defined as subjects that were found dead with no obvious signs of infection, trauma, or intervention during daily monitoring. Mortality risk for each allele was determined by comparing survival scores of cohorts aged upto 24 months. Overall, females had a greater risk than males, and survival probabilities were lowest in both sexes for animals expressing both LOAD risk alleles (Supplementary Figure 5).

### PET Identified Age- and Genotype-Dependent Differences in Glycolysis and Tissue Perfusion

**Figure 3:**
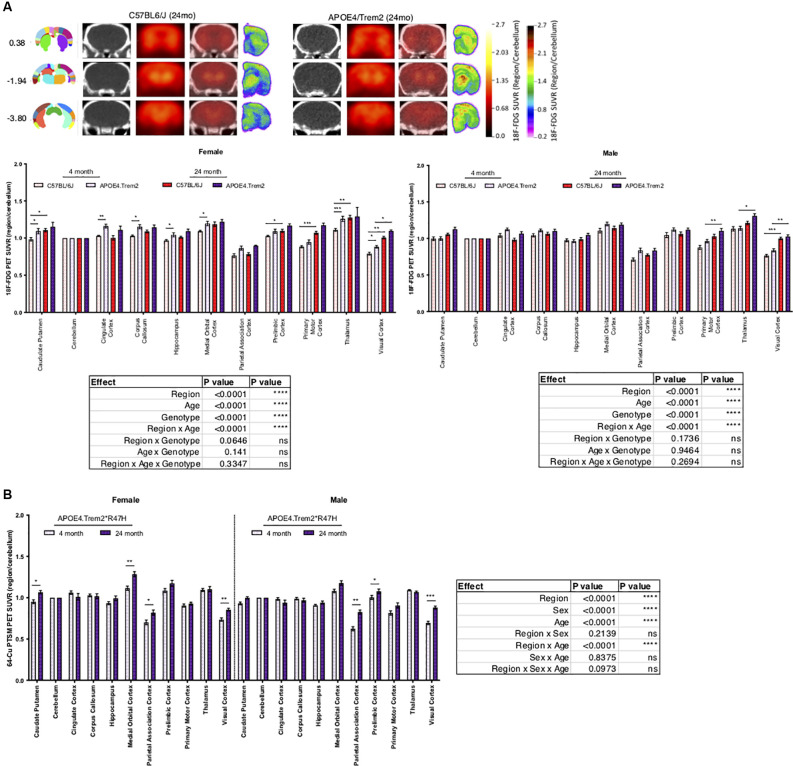
Increases in regional glucose metabolism due to expression of APOE4 and Trem2*R47H. Positron emission tomography (PET; red scale) of radioactive 18F-FDG marker was used to measure tissue glucose uptake, guided by computed tomography (CT; black and white) mapping to brain regions of interest, indicated by bregma coordinates (far left) **(A)**. PET-CT of 64-Cu PTSM of female and male B6.APOE4.Trem2*R47H mice revealed age-driven increases in brain perfusion **(B)**. Post-mortem autoradiography of coronal brain tissue is represented in **(A)** (rainbow; far right). Genotype- and age-dependent differences determined by three-way ANOVA. Factor effects and effect interaction are displayed in tables. *p < 0.05; **p < 0.01; ***p < 0.001. All alleles expressed were homozygous.

In an effort to understand the role of risk alleles on regional glycolysis and tissue perfusion, translationally relevant regional measures were acquired *via* 18F-FDG and 64Cu-PTSM PET and autoradiography, respectively (Figure 3). By 12 months glycolysis was altered in key brain regions associated with sensory integration, cognition, vision, and motor function in B6.*APOE4* and B6.APOE4.Trem2*R47H mice, when compared with controls (Table 3; Supplementary Figure 6), and were confirmed *via* post-mortem autoradiography, which has a 40-fold greater resolution than PET. As expected, these changes were greater in number of regions and magnitude of change in female mice when compared to males (Supplementary Figures 6B,C). These changes were similarly observed through time, where female mice showed significantly altered glycolysis at 4, 8, and 12 months, while male mice largely showed a hypoglycolytic phenotype at 8 months, that was virtually mitigated by 12 months (Supplementary Figure 6). Since these risk alleles can alter metabolic functionality and neuroinflammatory-driven tissue perfusion in an independent manner, we quantitatively measured changes in regional tissue perfusion *via* 64Cu-PTSM PET/MRI and confirmed this *via* autoradiography. Brain perfusion was significantly lower in regions associated with sensory integration, cognition, vision, and motor function in both sexes by 12 months and confirmed *via* post-mortem autoradiography (Supplementary Figure 7). Interestingly, these changes were manifested temporally, with the greatest reductions occurring across genotypes and regions in both sexes at 4 months (Supplementary Figure 7). Unlike glycolysis, these changes were largely resolved by 8 months in female mice, while males continued to show a regional reduction in perfusion at this same age. In 24-month-old animals, APOE4.Trem2*R47H had increased glycolysis compared to C57BL/6J in most brain regions, a trend that was also evident in young cohorts and in both sexes (Figure 3A). Age-related increases in brain perfusion were further confirmed in 24 months B6.APOE4.Trem2*R47H mice (Figure 3B).

**Table 3:** *In vivo* imaging study population.

Mouse models	4M		8M		12M	
	Male	Female	Male	Female	Male	Female
**C57BL/6J**	8	9	12	11	9	10
**APOE4 KI**	10	10	10	12	12	10
TREM2*R47H	10	12	12	8	12	11
APOE4. TREM2* R47H	12	10	10	12	10	10

### Biochemical and Neuropathological Effects of APOEε4 and Trem2*R47H Alleles

**Figure 4:**
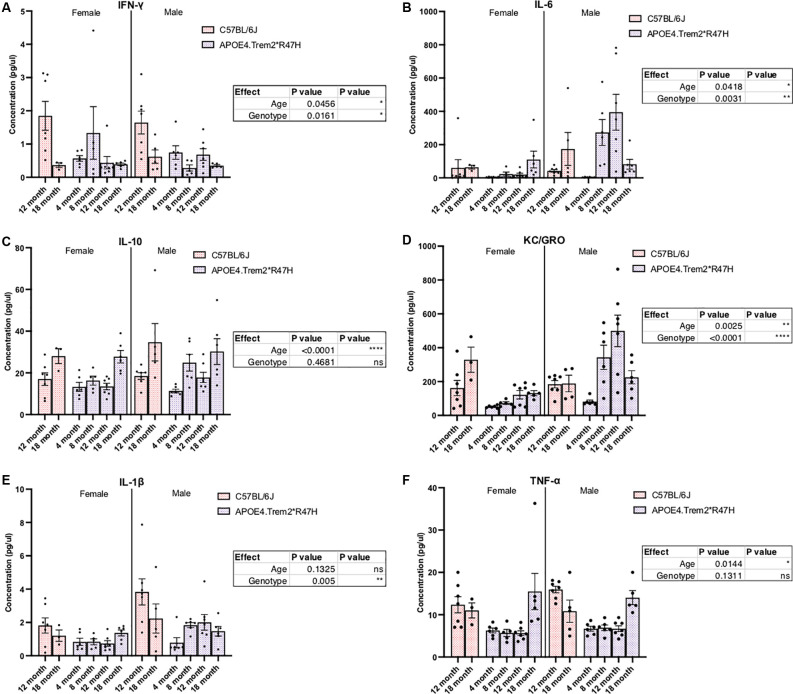
Levels of immunomodulatory cytokines in plasma are significantly different due to increased age and combined expression of APOE4 and Trem2*R47H alleles. IFN-gamma (IFN-γ), IL-1beta, IL-10, IL-6, and KC/GRO (CXCL1) levels were measured by multiplex immunoassays of blood plasma from C57BL/6J and B6.APOE4.Trem2R47H mice **(A–F)**. Genotype- and age-dependent differences determined by two-way ANOVA. Factor effects displayed in tables. *p < 0.05; **p < 0.01; ***p < 0.001. All alleles expressed were homozygous.

Confirmation of protein expression levels in brain tissue was confirmed for alleles encoding human APOE4 and mouse TREM2 carrying the R47H mutation (Supplementary Figure 8). Similar to reports of R47H variant-mediated reduction in *Trem2* transcript levels (Logsdon et al., 2019), TREM2 protein levels in the brains of these animals were also decreased. However, instead of a near knock-out of all TREM2 that has been reported previously (Logsdon et al., 2019), levels fell by approximately 50% in Trem2*R47H animals compared to C57BL/6J (Supplementary Figures 8A,B). We have previously shown APOE4 protein levels are similar to endogenous mouse APOE (Foley et al., 2020) and expression of APOE4 appeared similar between male and female APOE4.Trem2*R47H mice (Supplementary Figure 8C). Additional molecular characterization of these animals showed both age- and genotype-driven differences in levels of cytokines present in the blood (Figure 4) and brain (Supplementary Figure 9; Table 4). Compared to similarly aged C57BL/6J and younger APOE4.Trem2*R47H cohorts, 18 month B6.APOE4.Trem2*R47H mice showed strong differences in circulating concentrations of immunomodulatory cytokines (Figure 4). IL-6 and KC/GRO concentrations were highest in B6.APOE4.Trem2*R47H brain tissue at 8 months, while blood plasma concentrations continued to increase with age in those mice (Supplementary Figures 9B,D,E). On multiple occasions, a trend appeared to suggest increased cytokine concentrations in mice expressing mutated allele *Trem2*.

**Table 4:** Blood and brain tissue cytokine and metabolomic study population.

Mouse models	4M		8M		12M		24M	
	Male	Female	Male	Female	Male	Female	Male	Female
**C57BL/6J**	17	13	12	6	10	11	15	13
**APOE4 KI**	11	10	11	12	13	14	10	10
TREM2*R47H	12	11	11	11	12	10	13	13
APOE4.TREM2*R47H	10	10	11	11	8	5	8	5

**Figure 5:**
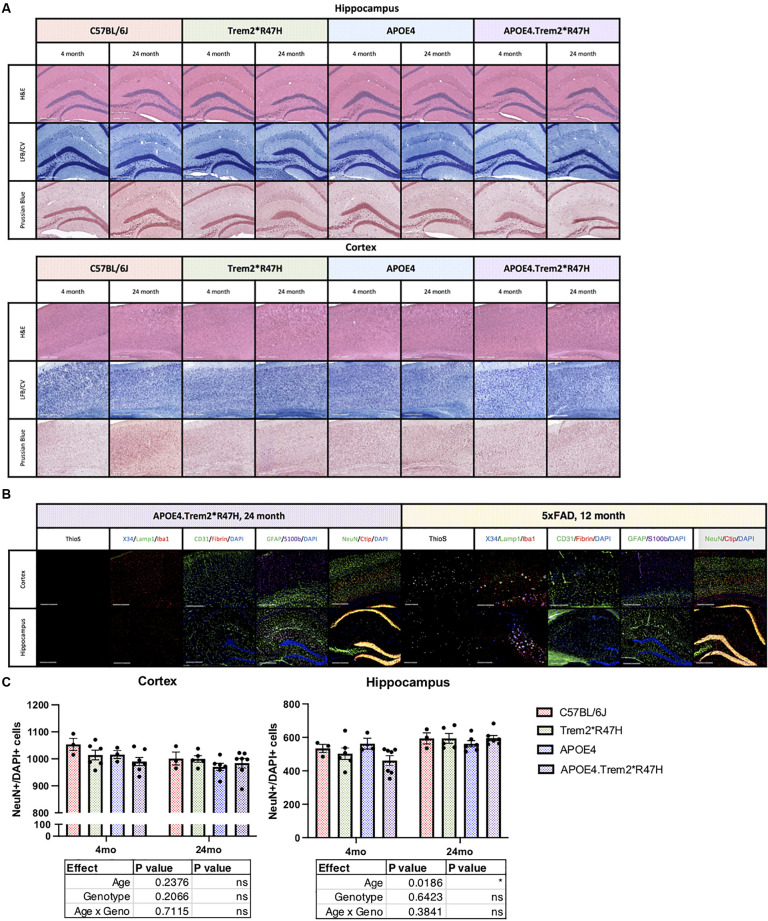
Neuropathological analysis of cortex and hippocampus. Hematoxylin and eosin (H&E), luxol fast blue/cresyl violet (LFB/CV), and Prussian blue staining tissue staining protocols **(A)** to identify anatomical changes in the cortex and hippocampus of young and aged mice. Thioflavin S (Thios) and four combinations of known neuropathological markers of LOAD in aged mice, 5xFAD included as amyloidogenic positive control **(B)**. Neuropathological analysis of cortex and hippocampus. Neuron (NeuN) cell populations were quantified in panel **(C)**. Age- and genotype-dependent differences determined by two-way ANOVA. Factor effects and effect interaction displayed in tables. *p < 0.05; **p < 0.01; ***p < 0.001. All alleles expressed were homozygous.

Neuropathological features of AD were then investigated by hematoxylin and eosin (H&E, structure) and luxol fast blue/cresyl violet (LFB/CV, myelin) staining but did not reveal any gross anatomical changes to tissue architecture or myelin (Figure 5A). Brain sections were also imaged *via* immunofluorescence and included neuritic plaque-reactive-microglia (X34/Lamp1/Iba1), vascular leakage (CD31/Iba1/Fibrin), and ThioflavinS (amyloid plaques and neurofibrillary tau tangles; Figure 5B). No gross abnormalities in cell counts (Figure 5C), nor additional neuropathological features were observed in 24-month B6.APOE4.Trem2*R47H mice. We focused particularly on the cortex and hippocampus where episodic memory (hippocampus) and memory behavior (cortex) are regulated. Amyloid plaques and hyperphosphorylated Tau were not observed in mice of any genotype at any age.

### Transcriptional Profiling Revealed Individual and Synergistic Effects of APOEε4, Trem2*R47H, and Age

**Figure 6:**
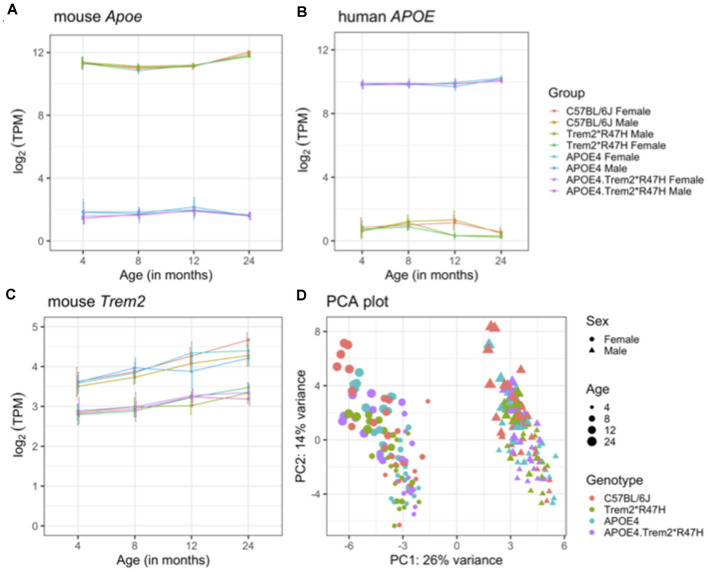
Overview of brain transcriptome. Expression levels of mouse *Apoe* **(A)**, human *APOE* **(B)**, and mouse *Trem2* **(C)** genes in the B6.*APOE4*, B6.Trem2*R47H, B6.APOE4.Trem2*R47H and C57BL/6J mice at 4, 8, 12, and 24 months in both sexes. Principal component analysis (PCA) of RNA-Seq transcriptomics data from all 234 samples **(D)**. The percent of variation explained by each principal component is displayed on the corresponding axis. Female and male samples are represented as circles and triangles, respectively. Genotypes are shown by different colors and the increasing size of points correspond to the increasing age of mice (4, 8, 12, and 24 months respectively). All alleles expressed were homozygous.

Brain hemispheres from 4 and 24-month male and female B6.APOE4.Trem2*R47H mice and single genotype and C57BL/6J controls were assessed using RNA-seq (see “Materials and Methods” section; Table 5). The transcriptomic analysis measured the expression levels (log-transformed TPM counts) of mouse *Apoe*, *Trem2*, and human *APOE* genes across all mouse models (Figures 6A–C). We observed higher expression of human APOE gene in mice carrying humanized *APOEε4* (*B6.APOE4* and B6.APOE4.Trem2*R47H mice), whereas mouse *Apoe* gene was highly expressed in B6 and Trem2*R47H mice (Figures 6A,B). As expected based on protein levels (Supplementary Figures 7A,B), expression of *Trem2* was significantly reduced (*p* < 0.05) in B6.Trem2*R47H and B6.APOE4.Trem2*R47H compared to age-matched B6 (Figure 6C), an effect likely caused by a novel effector splice site and truncation introduced by the R47H mutation. Furthermore, the expression level of *Trem2* increased with age across all mouse models, but no such patterns were observed in the expression levels of mouse *Apoe* and human *APOE* genes (Figures 6A,B). In addition, there was a lower expression of *Trem2* in B6.APOE4.Trem2*R47H compared to B6.Trem2*R47H mice at an advanced age (24 months), suggesting expression of *Trem2* might be suppressed by *APOE*ε4. Next, principal component analysis (PCA) identified two distinct clusters corresponding to male and female samples separated along with the first principal component (26% of total variance), suggesting sex-specific differences are profound in mice (Figure 6D). Analysis of samples from different age groups revealed a gradient of discrimination along with the second principal component (14% of total variance; Figure 6D), implying the presence of age-dependent molecular changes in the brain transcriptomes.

**Table 5:** RNA-seq study population.

Mouse models	4M		8M		12M		24M	
	Male	Female	Male	Female	Male	Female	Male	Female
**C57BL/6J**	1	1	6	6	6	6	7	6
**APOE4 KI**	13	12	6	6	4	5	5	6
TREM2*R47H	12	12	6	6	6	6	6	3
APOE4.TREM2*R47H	10	12	5	6	8	5	7	6

**Figure 7:**
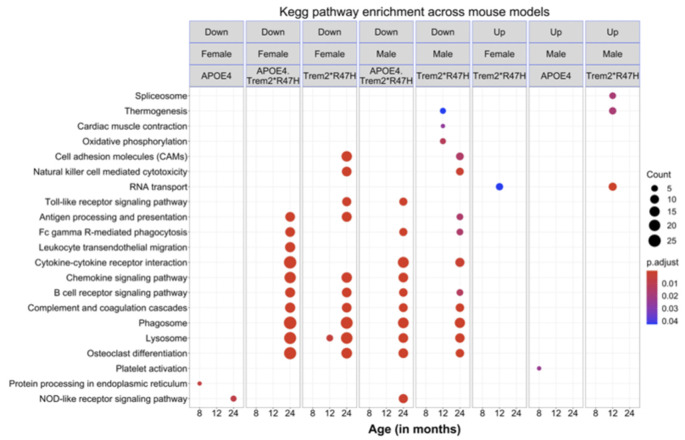
KEGG pathways enrichment analysis. Significantly enriched KEGG pathways (*p* < 0.05) in the downregulated and upregulated list of genes across mouse models at different ages for both sexes. All alleles expressed were homozygous.

To identify molecular effects of the LOAD risk genes, we performed pairwise differential analysis between each genotype (B6.*APOE4*, B6.Trem2*R47H or B6.APOE4.Trem2*R47H) and age- and sex-matched B6 controls. At an early age (4 months), only a few genes were differentially expressed (DEG; *p* < 0.05) for all genotypes for both sexes (Supplementary Figure 10A), and no KEGG pathways were enriched. At 8 months of age, there were 32 DEGs (3 upregulated, 29 downregulated; *p* < 0.05) in male B6.Trem2*R47H mice, and 11 DEGs (2 upregulated, 9 downregulated; *p* < 0.05) in female B6.Trem2*R47H mice (Supplementary Figure 10A). KEGG Pathway analysis identified enrichment of genes involved in immune-related pathways such as “complement and coagulation cascades” and “staphylococcus aureus infection” in the downregulated DEGs in female B6.Trem2*R47H mice (Figure 7). In 8 months old B6.*APOE4* mice, a total of 145 genes were significantly differentially expressed (42 upregulated, 103 downregulated; *p* < 0.05) in male mice, whereas a total of 25 genes were differentially expressed (11 upregulated, 14 downregulated; *p* < 0.05) in female mice (Supplementary Figure 10A). Pathway enrichment analysis of upregulated genes in male B6.*APOE4* mice identified enrichment of “platelet activation” pathway, whereas downregulated genes in female B6.*APOE4* mice were enriched for “protein processing in endoplasmic reticulum” pathway (Figure 7). No KEGG pathways were enriched for downregulated DEGs in male *B6.APOE4* mice and upregulated DEGs in female *B6.APOE4* mice. DEGs in male B6.APOE4.Trem2*R47H (7 upregulated, 32 downregulated), and female B6.APOE4.Trem2*R47H mice (2 upregulated, 1 downregulated; *p* < 0.05; Supplementary Figure 10, Figure 7) were not enriched in any KEGG pathway.

At 12 months of age, there were a total of 206 DEGs (118 upregulated, 88 downregulated) in male B6.Trem2*R47H mice and 285 DEGs (113 upregulated, 172 downregulated; *p* < 0.05) in female B6.Trem2*R47H mice. The upregulated DEGs in male Trem2*R47H mice were enriched in “RNA transport” and “spliceosome” pathways (Figure 7, Supplementary Figure 10), while genes in the “oxidative phosphorylation” pathway were downregulated in male B6.Trem2*R47H mice (Figure 7, Supplementary Figure 10). Upregulated and downregulated DEGs in female B6.Trem2*R47H mice were enriched for “RNA transport” and “lysosome” pathways respectively (Figure 7, Supplementary Figure 10). There were very few DEGs in *B6.APOE4* (1 in male, 3 in female) and B6.APOE4.Trem2*R47H mice (2 in male, 5 in female; Supplementary Figure 10) at this age and therefore no enrichment of KEGG Pathways.

At 24 months old, there were a total of 144 DEGs (15 upregulated, 129 downregulated) in the male B6.Trem2*R47H mice and 748 DEGs (359 upregulated, 389 downregulated; *p* < 0.05) in the female B6.Trem2*R47H mice. At this age, B6.APOE4.Trem2*R47H mice showed a greater number of DEGs (*p* < 0.05) in both male (24 upregulated, 197 downregulated) and female mice (83 upregulated, 400 downregulated) compared to at younger ages (4–12 months; Supplementary Figure 10), suggesting that the most dramatic transcriptional changes arise between 12 and 24 months. We also found substantial overlap in downregulated DEGs between B6.Trem2*R47H and B6.APOE4.Trem2*R47H mice for both sexes (Supplementary Figure 10). This suggests that the Trem2*R47H allele is the major driving force in age-dependent transcriptional changes in B6.APOE4.Trem2*R47H mice. Downregulated DEGs were enriched in multiple AD-related pathways such as “lysosome”, “osteoclast differentiation”, “phagosome”, “antigen processing and presentation”, cytokine-cytokine receptor interaction’, and “complement and coagulation cascades” in both 24 months old B6.Trem2*R47H and B6.APOE4.Trem2*R47H mice (Figure 7, Supplementary Figure 10). In B6.*APOE4* mice, we observed only two DEGs (1 upregulated, 1 downregulated) in male and 24 DEGs (1 upregulated, 23 downregulated) in female mice. Downregulated genes in the B6.*APOE4* female mice were enriched in “NOD-like receptor signaling” pathway (Figure 7, Supplementary Figure 10). We did not observe any enriched KEGG pathways in the upregulated list of genes across all genotypes at 24 months.

### Home Cage Voluntary Wheel Running Increased Oxidative Phosphorylation Pathway in APOE4.Trem2*R47H Mouse Brains

In the human population, a sedentary lifestyle is correlated with an increased risk of LOAD (de Rezende et al., 2014; Fenesi et al., 2017; Yan et al., 2020). Therefore, to determine if physical activity influenced transcriptional changes in B6.APOE4.Trem2*R47H mice, a running wheel was provided in the home cage of 22-month-old male mice for 2 months. Brain tissue from these animals was profiled by RNA-seq. A total of 292 DEGs (108 upregulated, 184 downregulated) were identified in the running B6.APOE4.Trem2*R47H mice compared to 24 months old B6 male mice. Enrichment analysis identified multiple enriched KEGG pathways such as “oxidative phosphorylation”, “thermogenesis”, and “retrograde endocannabinoid signaling” in the upregulated list of genes, whereas immune system associated pathways were enriched in the downregulated list of genes (Supplementary Figure 11A). To identify the effect of exercise, running B6.APOE4.Trem2*R47H male mice were compared with sedentary 24-month-old B6.APOE4.Trem2*R47H male mice and there was a total of 600 DEGs (312 upregulated, and 288 downregulated). Upregulated DEGs were enriched in pathways such as “oxidative phosphorylation” and “Ribosome” (Supplementary Figure 11A). The expression of these upregulated DEGs enriched for oxidative phosphorylation showed reduced expression in age- and sex-matched *B6.APOE4* and B6.Trem2*R47H compared to running B6.APOE4.Trem2*R47H mice (Supplementary Figure 11B). Finally, the expression of the upregulated DEGs associated with the oxidative phosphorylation pathway was assessed in transcriptional data from AMP-AD. Reduced expression of these running signature genes was observed in AD cases compared to controls across multiple brain regions such as parahippocampal gyrus (PHG) and frontal pole brain regions (FP; Supplementary Figure 11C). This suggests that exercise induces beneficial effects on health by increasing the expression of oxidative phosphorylation pathway genes that are down regulated across multiple brain regions in AD patients.

## Discussion

MODEL-AD was established in response to the many shortcomings of existing mouse models of AD. Aspects of human pathology have been replicated in mouse strains, most prominently the formation of beta-amyloid plaques *via* transgenic over-expression of brain-specific mutant human amyloid-beta precursor protein (APP), presenilin-1 (PS1), and/or microtubule-associated protein tau (MAPT) bearing familial Alzheimer’s disease (FAD) mutations (Bilkei-Gorzo, 2014). Legacy preclinical models rely heavily on alleles that overexpress transgenes, resulting in the removal or masking of important human-relevant biological interactions. These mouse strains have been invaluable for understanding the molecular and behavioral phenotypes of early-onset Alzheimer’s disease (EOAD) driven by the rapid and robust formation of plaques and tangles in the brain and correlating hyperactivity which is a confound of many cognitive behaviors. However, LOAD is ~20× more prevalent than EOAD and further implicates aging, inflammation, environmental, and many more genetic risk factors in disease development. APOE4.Trem2*R47H mice did not produce any severe phenotypes, even late into life, allowing a better understanding of the effect of AD risk factors in the context of aging (Supplementary Figure 5). As the heterogeneity of this disease becomes more appreciated, so is the importance of appropriate disease staging. Molecular targets of interest may only be available during particular evolving disease stages: debris (cell fragments, plaques, tangles, etc.) accumulates over time, and inflammation, interruption/loss of neuronal function all also change with disease progression. In light of the repeated shortcomings of “fit-for-all” therapies, efforts may be better directed at targeted therapies (Cummings et al., 2014, 2020; Safieh et al., 2019). Faithfully modeling a complex, polygenic disease will be aided by the creation of platform strains that carry multiple genetic risk factors to motivate with a scientific rationale rather than a grant-focused one. Of the candidate risk variants identified, expression of the ε4 allele of *APOE* and the R47H mutation in *TREM2* were identified as the strongest candidates for the initial development of a novel LOAD mouse strain. Introduction of the R47H mutation into *Trem2* resulted in the creation of a novel murine splice site yielding a decrease in approximately 50% of TREM2 protein (Supplementary Figures 8A,B), and a 20% decrease in *Trem2* transcript (Figure 6C). In the absence of amyloid plaque insult, the effects on microglia response due to the mutation and decreased expression are difficult to decipher. Similar models have shown similar decreases in R47H-mediated *Trem2* expression and function (Cheng et al., 2018; Cheng-Hathaway et al., 2018; Xiang et al., 2018). Efforts are ongoing to develop a *Trem2* allele expressing the full-length R47H risk factor at levels similar to wild-type *Trem2*. *APOEε4* is strongly associated with disease development and severity (Bu, 2009; Yamazaki et al., 2019) and at least one allele is present in approximately 65% of AD patients (Mayeux et al., 1998). Unfortunately, endogenous *Apoe* in mice does not express the isoform diversity seen from the *APOE* allele in humans. Insertion of humanized *APOE* alleles into mouse genomes has been a successful strategy to dissect the biology of APOE isoforms in mice (Knouff et al., 1999; Esquerda-Canals et al., 2017; Balu et al., 2019; Safieh et al., 2019; Lewandowski et al., 2020). The MODEL-AD *APOEε3* and *APOEε4* allelic series on a C57BL/6J background has subsequently be shown to consistently reproduce the biology presented in other *APOE* mouse models and most importantly, human patients (Knouff et al., 1999; Foley et al., 2020). Therefore, *APOE4* formed the basis for multiple platform strains that include: B6.APOE4.Trem2*R47H (for which we have provided the identifier “LOAD1”).

APOE binds to high-density lipoproteins to facilitate cholesterol and phospholipid transport to LDL receptors. As expected (Knouff et al., 1999; Maria Fe Lanfranco et al., 2020), lipoprotein levels differed compared to endogenous *Apoe*, mice expressing the humanized *APOEε4* allele showed decreased plasma lipoprotein levels across all time points (Supplementary Table 2). We observed an age-dependent decrease in glucose levels across both sexes and all genotypes (Figure 1L), an indication of increased frailty and aging that is common in aging-related-disease studies (Abdelhafiz et al., 2015). The contributions of metabolic and vascular factors are strongly implicated in disease progression, and how APOE or any other metabolic trait, *via* systemic pathways, can influence CNS function should be a continued focus for intervention (Zhao et al., 2020). The brain has one of the richest networks of blood vessels and is especially vulnerable (block or reduce blood flow, oxygen, and nutrients). For example, the respective influence of *APOEε4*, with and without Trem2*R47H expression, in regional changes in brain glycolytic metabolism (Murray et al., 2014; Figure 3, Supplementary Figure 6), tissue perfusion (Thambisetty et al., 2010; Roher et al., 2012; Figure 4, Supplementary Figure 7) observed in human AD remain under further study. Additional analyses of this *APOE4* allele have also revealed changes in cholesterol metabolism and transcriptional signatures in the brain compared to carriers of the *APOE3* allele (Foley et al., 2020).

Despite the heterogeneity of AD, age is the strongest risk factor in the human population. As an aging disease, monitoring and evaluating mouse models in relation to age is crucial for understanding the onset and progression of the disease over time. We selected timepoints that reflected different life stages of an adult mouse: Mature, by 3–6 months (beyond development but not yet affected by senescence); Middle-aged, 10–14 months (some senescent changes detected in some, not all, biomarkers of aging); and Aged, 18–24 months (senescent markers can be observed in all animals; Fox et al., 2007). Non-invasive testing of motor activity, behavior, and cognition have been shown to be reliable phenotypes for staging disease onset and trajectory (Sukoff Rizzo et al., 2018; Bogue et al., 2020). Aged mice equally displayed the expected trial-dependent increases in consecutive rotarod trials and decrease in total distance traveled over time in the open field assay, regardless of genotype (Supplementary Figure 3). Animal activity, measured by total distance traveled during open field assays (Figure 2, Supplementary Figure 2), also decreased equally with age. Similarly, home cage wheel running assays, which provide a more comprehensive activity phenotype than the 1-h open field test, also showed a decrease in activity levels during the active (dark phase; Sukoff Rizzo et al., 2018; Supplementary Figure 4). Interestingly, at 24 months some indications of increased activity during the active phase in animals carrying the *APOEε4* allele (both B6.*APOE4* and B6J.APOE4/Trem2*R47H) and day-time activity in only B6.Trem2*R47H mice suggesting a dominant phenotype produced by expression of APOEε4 (Supplementary Figures 4C,D, 10). Further consideration of genotype influences in behavioral, biometric, and molecular testing by statistical analysis was restricted by breeding strategy. Only littermate animals are considered for statistical comparisons, preventing C57BL6/J and B6.Trem2*R47H animal genotypes from inclusion within timepoints.

In the absence of additional environmental or genetic risk factors, B6.APOE4/Trem2*R47H mice did not display penetrant behavioral phenotypes beyond the expected aging-related changes but did exhibit decreased survival probabilities by 24 months (Supplementary Figure 5). Very few C57BL/6J mice succumbed during the 24-month aging process (<5%), whereas mortality was higher in mice expressing both LOAD alleles in both males (~20%) and females (~35%). Male mice expressing either allele alone had survival probabilities similar to C57BL/6J, whereas females with *APOEε4* or Trem2*R47H showed a mortality rate of ~20%. Therefore, it would seem that these two LOAD risk alleles show an equal and additive risk when expressed together, but in females, their interaction appears synergistic.

We investigated the molecular signatures in the brain transcriptomes of LOAD mouse models at different ages in both sexes. We identified age-dependent molecular changes associated with LOAD pathologies in mouse models. Introduction of the R47H mutation revealed a novel *Trem2* isoform identical to the primary transcript, but truncated by 119 bp from its start position in exon 2 (see “Materials and Methods” section; Kotredes, 2020). Expression of this novel isoform resulted in a decrease in both transcript and protein compared to wild-type *Trem2* carriers (Figure 6C, Supplementary Figures 8A,B). Despite the decrease in *Trem2* expression, mouse models carrying R47H mutation in the *Trem2* gene did not exhibit any significant transcriptional changes at a young age, in contrast, APOE4 mice exhibited significant changes only at 8 months of age. We further identified significant downregulation of genes associated with oxidative phosphorylation pathway in the 12 months old B6.Trem2*R47H mice, suggesting that oxidative phosphorylation could be prominent early feature for the onset of neurodegeneration/inflammation process. Subsequently, multiple immune-related processes were disrupted in 24 months old B6.Trem2*R47H and B6.APOE4.Trem2*R47H mice, supporting the profound relationship between aging, *Trem2*, and AD. Interestingly, at 12 months of age, we did not observe any significant transcriptional changes in B6.APOE4.Trem2*R47H mice compared to control mice, suggesting that the effect of *Trem2* gene is suppressed due to the presence of *APOEε4*. Similarly, when mouse models were compared with human co-expression modules, we observed a strong negative correlation between the B6.Trem2*R47H mice and immune-related human co-expression modules from multiple brain regions and this inflammatory response is dampened in the presence of *APOEε4* in the B6.APOE4.Trem2*R47H mice. Distinct mouse models showed concordance with distinct human co-expression modules reflecting a different transcriptional response driven by the human *APOEε4* and Trem2*R47H risk variants. We also observed age-dependent shift in co-expression patterns associated with LOAD pathologies. A strong negative correlation between co-expression modules associated with cell cycle and DNA repair was observed in the early-aged mouse B6.*APOE4* model, whereas advanced-aged B6.*APOE4* female mice showed a strong positive correlation with these co-expression modules. This overlap with human late-onset co-expression signatures early in life was observed for a number of different brain regions and was absent in Trem2*R47H knock-in mice. Furthermore, aged B6.Trem2*R47H mice showed a moderate overlap with several human neuronal co-expression modules enriched for genes that play an important role in synaptic signaling and myelination. At an advanced age, a strong correlation between the mouse models and immune-related human co-expression modules highlights the important role of the LOAD-associated *APOEε4* and *TREM2* R47H variant in Alzheimer’s related immune processes. Our experiments predict that *APOEε4* functions through the suppression of effects brought out by expression of the Trem2*R47H allele, displayed by the 455 genes that are upregulated by *TREM2^R47H^* but suppressed by *APOEε4* (Supplementary Figure 10). Our results mirror some emerging evidence that *APOEε4* suppresses Trem2*R47H in AD risk, that there are some suggestions that *APOEε4* carriers do not have increased AD risk with Trem2*R47H and Trem2*R47H only increases the risk on *APOEε3* carriers (Jendresen et al., 2017; Fitz et al., 2020). Additionally, we observe more differentially expressed genes at middle age than at a later age supporting evidence of an earlier aging phenotype than C57BL/6J mice, with a realignment of transcriptomes at later timepoints (McGeer et al., 1997; Zhao et al., 2020). We employed a weighted gene co-expression network analysis (WGCNA) used to identify modules of correlated genes. Each module was tested for differential expression by strain, then compared with human postmortem brain modules from the Accelerating Medicine’s Partnership for AD (AMP-AD) to determine the LOAD-related processes affected by each genetic risk factor (Logsdon et al., 2019; Pandey et al., 2019; Wan et al., 2020). This will be a useful tool in identifying differentially expressed genes correlated with molecular pathways tied to inflammation and identifying a mouse strain that exhibits a similar transcriptional signature to human patients with true neuroinflammation.

Amyloid plaque formation is a primary diagnostic measure of Alzheimer’s disease with both APOE and TREM2 linked to amyloid deposition (McGeer et al., 1997; Blennow et al., 2006; Bilkei-Gorzo, 2014; Kanekiyo et al., 2014; Jay et al., 2015; Cacace et al., 2016; Dourlen et al., 2019; Parhizkar et al., 2019). For example, TREM2 can bind amyloid, altering microglial function, linking the TREM2-APOE pathway directly to amyloid-driven disease progression (Zhao et al., 2018; Kober et al., 2020). Loss of functional *Trem2* in mice resulted in plaques that contained reduced amounts of APOE and promoted amyloidogenesis in mice by reducing microglial function (Parhizkar et al., 2019; McQuade et al., 2020) indicating that microglia, through TREM2 mediated signaling, can regulate APOE co-deposition around amyloid deposits. Further, TREM2 KO prevented infiltration of blood-derived myeloid cells and ameliorated plaque burden in *APP/PS1* mice (Jay et al., 2015) and disease-related mutations impair many of its functions (Kober et al., 2016). However, current amyloidogenic mouse models develop amyloid plaques at very young ages, within a few months (Supplementary Figure 7B), whereas in human patients the average age of AD onset is at older ages, ~80 years, potentially causing the disparity in therapeutic outcomes between mouse models and human patients. In the absence of amyloid deposition, many hallmarks of LOAD can be investigated for *APOEε4*- and *Trem2*-influenced effects that precede and may contribute to the onset of AD. However, current work is evaluating the effects of APOE and TREM2 risk alleles in the context of humanized Abeta. For instance, we are currently evaluating a novel B6.APOE4.Trem2*R47H.hAbeta (LOAD2) strain and in the process of incorporating humanized Tau (MAPT) alleles into forthcoming strains. These novel platforms or AD-sensitized strains (e.g., LOAD1, LOAD2, etc.) are being used to assess the contribution of additional genetic risk factors identified through genetic and genome-wide association approaches using LOAD1 as a platform strain. These include variations in genes commonly associated with AD including *ABCA7, PLCG2, CR1, BIN1*, and *SORL1*. The new strains are prioritized for extensive phenotyping using a primary screening approach centered on transcriptional profiling of nearly 800 genes known to be differentially expressed in human AD brains compared to unaffected controls (Preuss et al., 2020). Platform strains are also ideal for studying age-dependent effects of environmental risk factors, such as diet, as well as genetic context. Deeper analysis of APOE4.Trem2*R47H transcriptional data, *in vivo* imaging, and neuropathology samples are continuing and will be detailed in future publications. Amendments to the current phenotyping strategy are also in consideration to expand characterizations of the metabolome, proteome, and electrophysiology of LOAD animals. In subsequent studies utilizing new mouse strains, the utility of the APOE4.Trem2*R47H datasets will grow. Ultimately, strains carrying combinations of risk factors that more closely align with the human disease will be incorporated into the pre-clinical testing core of MODEL-AD to assess the potential of prioritized compounds to treat AD.

## Arrive Guidelines Statement

In accordance with ARRIVE (Animal Research: Reporting of *In Vivo* Experiments) guidelines, design and description of experimental animal cohorts are provided to ensure scientific rigor and reproducibility (Percie du Sert et al., 2020; https://arriveguidelines.org/).

## Data Availability Statement

The datasets presented in this study can be found in online repositories. The names of the repository/repositories and accession number(s) can be found in the article/Supplementary Material.

## Ethics Statement

The animal study was reviewed and approved by Animal Care and Use Committee at The Jackson Laboratory and Indiana University in accordance with guidelines set out in The Eighth Edition of the Guide for the Care and Use of Laboratory Animals. All euthanasia used methods were approved by the American Veterinary Medical Association.

## Author Contributions

KK and RP: data curation, formal analysis, investigation, methodology, and writing. AO: conceptualization, data curation, formal analysis, investigation, methodology, and writing. PL: formal analysis and investigation. DG, BL, and LM: investigation and methodology. HW: data curation, formal analysis, investigation, and methodology. AU: data curation, formal analysis, and investigation. RO’R, SO’R, CI, DB, MB, ZC, and KF: Investigation. SS and PT: conceptualization, data curation, investigation, methodology, and writing. GC: conceptualization, data curation, methodology, and writing. MS and BL: conceptualization, methodology, and writing. GH: conceptualization, investigation, methodology, and writing.

## Conflict of Interest

The authors declare that the research was conducted in the absence of any commercial or financial relationships that could be construed as a potential conflict of interest.

## Publisher’s Note

All claims expressed in this article are solely those of the authors and do not necessarily represent those of their affiliated organizations, or those of the publisher, the editors and the reviewers. Any product that may be evaluated in this article, or claim that may be made by its manufacturer, is not guaranteed or endorsed by the publisher.

## Footnotes

^1^ https://www.jax.org/strain/027894

^2^ https://www.jax.org/strain/027918

^3^ https://www.synapse.org/#!Synapse:syn11180450

^4^ https://adknowledgeportal.org

^5^ https://adknowledgeportal.org/DataAccess/Instructions

## Abbreviations

AD: Alzheimer’s disease
ADGC: Alzheimer’s Disease Genetics Consortium
ADNI: Alzheimer’s Disease Neuroimaging Initiative
ADSP: Alzheimer’s Disease Sequencing Project
AMP-AD: Accelerating Medicines Partnership—Alzheimer’s Disease
B6: C57BL/6J
BBB: Blood-brain barrier
CNS: Central nervous system
DEG: Differentially expressed genes
FP: frontal pole brain regions
HDL: high-density lipoproteins
IGAP: International Genomics of Alzheimer’s Project
LOAD: Late-onset Alzheimer’s disease
LDL: low-density lipoproteins
M^2^OVE-AD: Molecular Mechanisms of the Vascular Etiology of Alzheimer’s Disease
MRI: Magnetic resonance imaging
PET: Positron emission tomography
PHG: parahippocampal gyrus
ROS/MAP: Religious Orders Study and Memory and Aging Project.
