# Corrigendum: Uncovering Disease Mechanisms in a Novel Mouse Model Expressing Humanized APOEε4 and Trem2^*^R47H

**DOI:** 10.3389/fnagi.2022.857628

**Published:** 2022-02-07

**Authors:** Kevin P. Kotredes, Adrian Oblak, Ravi S. Pandey, Peter Bor-Chian Lin, Dylan Garceau, Harriet Williams, Asli Uyar, Rita O'Rourke, Sarah O'Rourke, Cynthia Ingraham, Daria Bednarczyk, Melisa Belanger, Zackary Cope, Kate E. Foley, Benjamin A. Logsdon, Lara M. Mangravite, Stacey J. Sukoff Rizzo, Paul R. Territo, Gregory W. Carter, Michael Sasner, Bruce T. Lamb, Gareth R. Howell

**Affiliations:** ^1^The Jackson Laboratory, Bar Harbor, ME, United States; ^2^Stark Neurosciences Research Institute, School of Medicine, Indiana University Bloomington, Indianapolis, IN, United States; ^3^Department of Medicine—Aging Institute, University of Pittsburgh School of Medicine, Pittsburgh, PA, United States; ^4^Sage Bionetworks, Seattle, WA, United States

**Keywords:** ApoE4, TREM2, mouse model, MODEL-AD, late-onset AD

An author name was incorrectly spelled as “Daria Bednarycek”. The correct spelling is “Daria Bednarczyk”.

The authors apologize for this error and state that this does not change the scientific conclusions of the article in any way. The original article has been updated.

## Publisher's Note

All claims expressed in this article are solely those of the authors and do not necessarily represent those of their affiliated organizations, or those of the publisher, the editors and the reviewers. Any product that may be evaluated in this article, or claim that may be made by its manufacturer, is not guaranteed or endorsed by the publisher.

