# Supplementary material for: Uncovering Disease Mechanisms in a Novel Mouse Model Expressing Humanized APOEε4 and Trem2*R47H
Source: Front Aging Neurosci. 2021 Oct 11;13:735524. doi: 10.3389/fnagi.2021.735524 (PMC8544520; doi:10.3389/fnagi.2021.735524)
Supplement: Supplementary file 1 [file Data_Sheet_1.pdf]

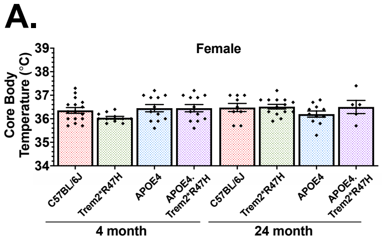

Effect	P value	P value
Age	0.0023	**
Genotype	0.5077	ns
Age x Geno	0.0723	ns

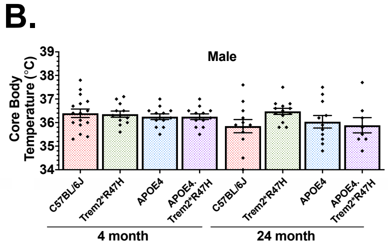

Effect	P value	P value
Age	<0.0001	****
Genotype	0.8504	ns
Age x Geno	0.2982	ns

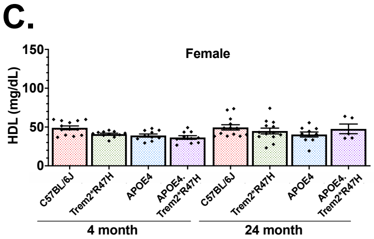

Effect	P value	P value
Age	0.0911	ns
Genotype	<0.0001	****
Age x Geno	0.1672	ns

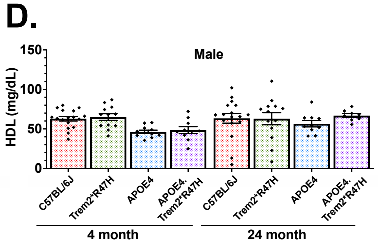

Effect	P value	P value
Age	0.1021	ns
Genotype	<0.0001	****
Age x Geno	0.0462	*

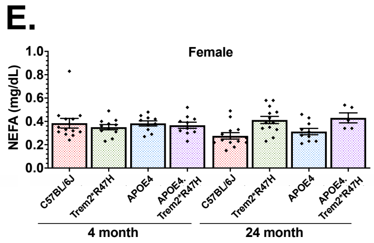

Effect	P value	P value
Age	<0.0001	****
Genotype	0.8779	ns
Age x Geno	0.0012	**

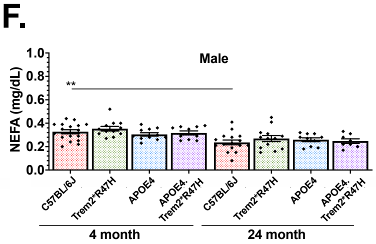

Effect	P value	P value
Age	<0.0001	****
Genotype	0.0853	ns
Age x Geno	0.0026	**

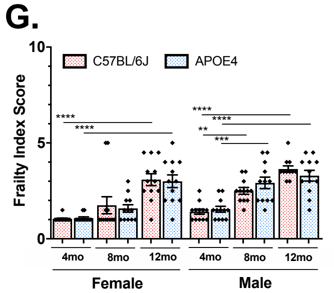

Effect	P value	P value
Age	<0.0001	****
Sex	<0.0001	****
Genotype	0.984	ns
Age x Sex	0.1441	ns
Age x Genotype	0.5395	ns
Sex x Genotype	0.6248	ns
Age x Sex x Genotype	0.4438	ns

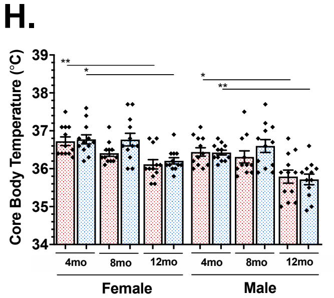

Effect	P value	P value
Age	<0.0001	****
Sex	0.0008	***
Genotype	0.0821	ns
Age x Sex	0.4018	ns
Age x Genotype	0.092	ns
Sex x Genotype	0.4772	ns
Age x Sex x Genotype	0.9326	ns

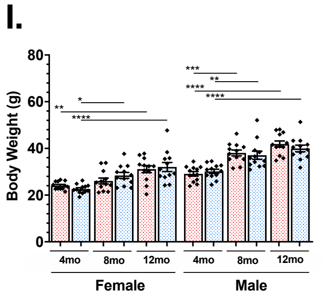

Effect	P value	P value
Age	<0.0001	****
Sex	<0.0001	****
Genotype	0.9822	ns
Age x Sex	0.0746	ns
Age x Genotype	0.797	ns
Sex x Genotype	0.4191	ns
Age x Sex x Genotype	0.2005	ns

Supplemental Figure 1. Biometric profiling of novel LOAD mouse strains in young and aged animals. Young and aged mice assessed by body temperature (A,B) and serum levels of non-fasted high-density lipoprotein (HDL) and non-esterified fatty acids (NEFA) (C-F). Littermate-controlled cohorts of young, old, and intermediate ages tested for measures of frailty due to age and homozygous expression of humanized *APOE4* allele (G-I). Age-dependent differences within genotype and sex determined by two- or three-way ANOVA. Factor effects and effect interaction displayed in tables. *p<0.05; **p<0.01; ***p<0.001. All alleles expressed were homozygous.

		Cumulative Frailty Index Score								Core Body Temperature (°C)								Animal Weight							
		Female				Male				Female				Male				Female				Male			
	Genotype	n=	Mean	Coeff. Variation	SEM	n=	Mean	Coeff. Variation	SEM	n=	Mean	Coeff. Variation	SEM	n=	Mean	Coeff. Variation	SEM	n=	Mean	Coeff. Variation	SEM	n=	Mean	Coeff. Variation	SEM
4 month	C57BL/6J	16	1.375	55.55%	0.1909	16	1.938	57.99%	0.2809	16	36.36	1.34%	0.1221	16	36.39	1.98%	0.1802	16	21.76	8.61%	0.4684	16	28.68	4.89%	0.3507
	Trem2*R47H	11	1.682	33.30%	0.1689	12	2.542	37.94%	0.2784	11	36.05	0.53%	0.05778	12	36.36	1.23%	0.1294	11	19.21	5.84%	0.3383	12	26.5	5.23%	0.4002
	APOE4	12	1.625	26.65%	0.125	12	2.875	40.70%	0.3378	12	36.45	1.48%	0.1555	12	36.25	1.16%	0.1215	12	22.25	7.71%	0.4949	12	28.32	10.49%	0.8576
	APOE4.<br>Trem2*R47H	10	1.5	60.86%	0.2887	11	2.636	31.84%	0.2531	12	36.45	1.48%	0.1555	12	36.25	1.16%	0.1215	12	22.25	7.71%	0.4949	12	28.32	10.49%	0.8576
8 month	C57BL/6J	18	2.111	38.58%	0.192	18	3.167	29.66%	0.2214	18	36.48	1.07%	0.09202	18	36.29	2.03%	0.1737	18	25.09	9.16%	0.5418	18	34.09	5.58%	0.4487
	Trem2*R47H	12	1.75	31.06%	0.1569	12	2.458	25.22%	0.179	12	36.21	1.13%	0.1184	12	36.06	1.38%	0.1433	12	22.25	3.90%	0.2503	12	26.97	9.27%	0.722
	APOE4	12	2.208	26.37%	0.1681	12	3.583	18.66%	0.193	12	36.5	0.90%	0.09535	12	36.05	1.95%	0.2024	12	22.66	13.79%	0.9023	12	31.32	11.53%	1.042
	APOE4.<br>Trem2*R47H	11	2.364	30.05%	0.2142	12	3.083	28.37%	0.2525	12	36.5	0.90%	0.09535	12	36.05	1.95%	0.2024	12	22.66	13.79%	0.9023	12	31.32	11.53%	1.042
12 month	C57BL/6J	21	5.048	16.55%	0.1823	23	5.043	12.65%	0.133	21	35.97	0.93%	0.07314	23	35.5	1.16%	0.08562	21	31.93	16.48%	1.148	23	41.31	8.02%	0.6906
	Trem2*R47H	11	3.136	31.96%	0.3022	13	5.038	11.07%	0.1546	11	36.24	1.44%	0.1568	13	35.36	1.17%	0.1147	11	25.47	16.86%	1.295	13	36.53	11.14%	1.128
	APOE4	10	2.75	33.47%	0.2911	11	5.273	18.17%	0.2889	10	36.13	1.05%	0.1202	11	35.62	1.43%	0.153	10	23.6	8.96%	0.6687	11	37.2	16.59%	1.861
	APOE4.<br>Trem2*R47H	10	2.71	24.50%	0.21	12	5.042	24.46%	0.356	10	36.13	1.05%	0.1202	11	35.62	1.43%	0.153	10	23.6	8.96%	0.6687	11	37.2	16.59%	1.861
24 month	C57BL/6J	9	5	43.59%	0.7265	12	6.125	22.59%	0.3995	9	36.48	1.44%	0.1746	12	35.85	2.71%	0.28	9	29.91	12.29%	1.225	12	34.9	19.76%	1.99
	Trem2*R47H	14	3.714	42.15%	0.4184	13	5.731	22.13%	0.3518	14	36.51	1.00%	0.09713	13	36.48	1.26%	0.1272	14	30.34	14.08%	1.142	13	40.68	15.90%	1.794
	APOE4	11	4.045	28.96%	0.3533	11	6.091	11.50%	0.2113	11	36.2	1.19%	0.13	11	36.04	2.44%	0.2647	11	32.14	14.00%	1.356	11	37.29	10.50%	1.18
	APOE4.<br>Trem2*R47H	5	4.5	33.33%	0.6708	8	6.313	13.35%	0.2979	5	36.5	1.71%	0.2793	8	35.89	2.53%	0.3215	5	32.4	13.44%	1.948	8	36.24	13.32%	1.706

Supplemental Table 1. Complete data set of frailty assay measures of LOAD strains by age. Individual animals from cross-sectional cohorts housed to the ages indicated were assessed by 27 physical measures to provide a Cumulative Frailty Index Score. Animals were also measured for core body temperature and weight. All alleles expressed were homozygous.

Total Cholesterol (mg/dL)

Age	Genotype	Female n=	Female Mean	Female Coeff. Variation	Female SEM	Male n=	Male Mean	Male Coeff. Variation	Male SEM
4 month	C57BL/6J	13	66.23	17.70%	3.251	17	82.94	19.68%	3.96
	Trem2*R47H	11	53.45	11.08%	1.786	12	82.58	22.02%	5.249
	APOE4	10	55.4	14.52%	2.544	11	57.45	15.10%	2.616
	APOE4. Trem2*R47H	10	52.5	19.18%	3.184	10	58.6	27.75%	5.143
8 month	C57BL/6J	6	80.17	19.75%	6.462	12	81.92	16.12%	3.813
	Trem2*R47H	11	58.64	12.11%	2.142	11	72.27	18.52%	4.036
	APOE4	12	49.5	15.80%	2.258	11	64	17.73%	3.422
	APOE4. Trem2*R47H	11	55.55	25.30%	4.237	11	71.55	23.44%	5.057
12 month	C57BL/6J	11	75.27	16.37%	3.715	10	87.9	15.04%	4.181
	Trem2*R47H	10	67.6	22.28%	4.764	12	93.92	17.46%	4.733
	APOE4	14	51.29	11.35%	1.556	13	56.31	18.43%	2.879
	APOE4. Trem2*R47H	3	53.67	5.99%	1.856	8	61.13	14.00%	3.026
24 month	C57BL/6J	13	71	28.76%	5.664	15	88.47	25.69%	5.869
	Trem2*R47H	13	64.69	31.49%	5.649	13	89.08	35.66%	8.809
	APOE4	10	60	32.06%	6.083	10	74	18.65%	4.364
	APOE4. Trem2*R47H	5	73	28.29%	9.236	8	90.88	33.19%	10.66

LDL (mg/dL)

Age	Genotype	Female n=	Female Mean	Female Coeff. Variation	Female SEM	Male n=	Male Mean	Male Coeff. Variation	Male SEM
4 month	C57BL/6J	13	4.784	29.30%	0.3887	17	3.368	51.05%	0.417
	Trem2*R47H	11	4.555	12.96%	0.178	12	2.041	22.38%	0.1319
	APOE4	10	1.244	34.83%	0.137	11	0.6791	52.92%	0.1084
	APOE4. Trem2*R47H	10	1.353	35.95%	0.1538	10	0.567	39.15%	0.07019
8 month	C57BL/6J	6	5.435	27.64%	0.6134	12	3.712	54.96%	0.5889
	Trem2*R47H	11	4.421	15.95%	0.2125	11	1.905	23.06%	0.1324
	APOE4	12	1.375	34.94%	0.1387	11	0.6582	44.12%	0.08755
	APOE4. Trem2*R47H	11	1.179	41.38%	0.1471	11	0.5991	26.80%	0.0484
12 month	C57BL/6J	12	3.858	31.94%	0.3558	11	2.55	15.34%	0.118
	Trem2*R47H	11	5.204	28.39%	0.4454	13	2.761	49.52%	0.3792
	APOE4	13	0.7246	44.08%	0.0886	14	0.3929	75.55%	0.07932
	APOE4. Trem2*R47H	4	1.013	57.61%	0.2916	8	0.5313	59.98%	0.1127
24 month	C57BL/6J	13	5.576	36.69%	0.5675	14	4.811	44.53%	0.5725
	Trem2*R47H	13	3.378	83.16%	0.7791	12	4.32	58.79%	0.7331
	APOE4	8	1.293	59.70%	0.2728	10	1.024	73.53%	0.2381
	APOE4. Trem2*R47H	4	0.8675	38.43%	0.1667	7	0.3329	133.09%	0.1674

HDL (mg/dL)

Age	Genotype	Female n=	Female Mean	Female Coeff. Variation	Female SEM	Male n=	Male Mean	Male Coeff. Variation	Male SEM
4 month	C57BL/6J	13	49.04	17.85%	2.427	17	62.94	18.62%	2.841
	Trem2*R47H	11	40.9	9.43%	1.163	12	65.09	22.11%	4.154
	APOE4	10	39.07	16.36%	2.021	11	46.29	15.39%	2.148
	APOE4. Trem2*R47H	10	36.54	19.90%	2.3	10	48.61	27.79%	4.272
8 month	C57BL/6J	6	56	24.70%	5.647	12	60.25	16.47%	2.865
	Trem2*R47H	11	41.14	17.10%	2.122	11	56.25	17.65%	2.993
	APOE4	12	34.63	22.43%	2.242	11	50.42	20.76%	3.155
	APOE4. Trem2*R47H	10	40.15	30.99%	3.935	10	56.81	26.57%	4.774
12 month	C57BL/6J	12	56.44	16.36%	2.665	11	70.79	20.51%	4.377
	Trem2*R47H	11	51.31	17.45%	2.7	13	73.06	17.20%	3.485
	APOE4	14	37.7	16.57%	1.67	14	45.96	19.08%	2.344
	APOE4. Trem2*R47H	4	39.98	3.60%	0.7204	9	50.72	16.56%	2.8
24 month	C57BL/6J	13	49.53	24.92%	3.423	17	63.38	39.15%	6.018
	Trem2*R47H	13	44.88	29.78%	3.708	13	62.94	43.50%	7.593
	APOE4	10	40.35	25.04%	3.195	10	56.59	22.69%	4.06
	APOE4. Trem2*R47H	5	47.64	29.38%	6.26	8	66.89	10.55%	2.495

Glucose (mg/dL)

Age	Genotype	Female n=	Female Mean	Female Coeff. Variation	Female SEM	Male n=	Male Mean	Male Coeff. Variation	Male SEM
4 month	C57BL/6J	13	491.6	16.08%	21.93	17	544.3	18.24%	24.07
	Trem2*R47H	11	446.5	20.24%	27.25	12	558.3	15.78%	25.44
	APOE4	10	469.6	20.96%	31.12	11	549.9	18.18%	30.14
	APOE4. Trem2*R47H	10	456	20.26%	29.22	10	556.5	27.00%	47.51
8 month	C57BL/6J	6	539.5	25.07%	55.21	12	548.2	17.88%	28.3
	Trem2*R47H	11	444.9	15.03%	20.17	11	543.3	18.33%	30.03
	APOE4	11	438.7	19.20%	25.4	11	621.6	23.97%	44.93
	APOE4. Trem2*R47H	11	487.1	16.85%	24.75	12	548.2	17.88%	28.3
12 month	C57BL/6J	12	535.1	16.00%	24.72	11	548.5	26.20%	43.33
	Trem2*R47H	11	472	19.56%	27.84	13	572.6	27.03%	42.93
	APOE4	15	426.1	19.48%	21.43	14	528.4	17.79%	25.13
	APOE4. Trem2*R47H	4	431	10.96%	23.63	9	599.9	26.28%	52.56
24 month	C57BL/6J	13	347.1	18.85%	18.14	17	294.1	28.96%	20.66
	Trem2*R47H	13	381.8	15.87%	16.8	13	300.7	43.59%	36.36
	APOE4	10	303.5	18.33%	17.59	10	336.6	22.70%	24.16
	APOE4. Trem2*R47H	5	342.2	13.09%	20.04	8	360.4	16.26%	20.72

NEFA (mg/dL)

Age	Genotype	Female n=	Female Mean	Female Coeff. Variation	Female SEM	Male n=	Male Mean	Male Coeff. Variation	Male SEM
4 month	C57BL/6J	13	0.3846	38.98%	0.04158	17	0.3271	22.54%	0.01788
	Trem2*R47H	11	0.3509	20.96%	0.02218	12	0.3533	19.09%	0.01948
	APOE4	10	0.383	17.97%	0.02176	11	0.3045	16.04%	0.01473
	APOE4. Trem2*R47H	10	0.367	22.73%	0.02638	10	0.317	17.02%	0.01707
8 month	C57BL/6J	6	0.3267	40.32%	0.05377	12	0.2892	23.15%	0.01932
	Trem2*R47H	11	0.2364	26.91%	0.01918	11	0.1836	20.56%	0.01138
	APOE4	12	0.275	22.50%	0.01786	11	0.2536	25.57%	0.01955
	APOE4. Trem2*R47H	11	0.2836	24.99%	0.02137	11	0.2455	21.89%	0.0162
12 month	C57BL/6J	12	0.2225	60.00%	0.03854	11	0.2836	21.63%	0.0185
	Trem2*R47H	11	0.1845	44.31%	0.02466	13	0.1954	32.61%	0.01767
	APOE4	15	0.2733	42.05%	0.02968	14	0.2814	39.44%	0.02967
	APOE4. Trem2*R47H	4	0.175	9.90%	0.00866	9	0.2367	24.46%	0.01929
24 month	C57BL/6J	13	0.2762	35.59%	0.02726	16	0.2363	33.75%	0.01993
	Trem2*R47H	13	0.4123	27.26%	0.03118	13	0.2692	33.92%	0.02533
	APOE4	10	0.313	27.65%	0.02737	10	0.259	20.14%	0.0165
	APOE4. Trem2*R47H	5	0.43	22.31%	0.0429	8	0.2475	22.42%	0.01962

Triglycerides (mg/dL)

Age	Genotype	Female n=	Female Mean	Female Coeff. Variation	Female SEM	Male n=	Male Mean	Male Coeff. Variation	Male SEM
4 month	C57BL/6J	13	114.3	31.45%	9.971	17	95.41	47.51%	10.99
	Trem2*R47H	11	76.45	20.55%	4.737	12	90.33	42.04%	10.96
	APOE4	10	109.5	29.05%	10.06	11	94.64	48.15%	13.74
	APOE4. Trem2*R47H	10	104	38.64%	12.71	10	86.4	32.77%	8.953
8 month	C57BL/6J	6	107.3	25.66%	11.24	12	82.67	36.13%	8.622
	Trem2*R47H	11	81.73	35.40%	8.724	11	97.45	31.00%	9.108
	APOE4	12	103.4	22.94%	6.848	11	112.4	17.64%	5.976
	APOE4. Trem2*R47H	11	109.4	24.24%	7.992	11	118.3	31.06%	11.08
12 month	C57BL/6J	12	95.17	27.64%	7.595	11	91.82	46.99%	13.01
	Trem2*R47H	11	87.09	29.82%	7.829	13	105.6	36.34%	10.64
	APOE4	15	86.93	21.51%	4.829	14	77.21	34.56%	9.208
	APOE4. Trem2*R47H	4	111.5	2.97%	1.658	9	139.7	60.38%	28.11
24 month	C57BL/6J	13	59.62	35.97%	5.947	17	54.24	51.90%	6.827
	Trem2*R47H	12	68.33	27.51%	5.426	13	52.08	28.81%	4.161
	APOE4	10	77.4	42.84%	10.49	10	63.3	36.30%	7.266
	APOE4. Trem2*R47H	4	73	17.44%	6.364	8	52.13	34.66%	6.388

Supplemental Table 2. Complete data set of non-fasted blood serum biochemistry analysis from novel LOAD strains by age. Blood serum of individual animals from cross-sectional cohorts housed to the ages indicated were measured for a panel of analytes. All alleles expressed were homozygous.

		Latency to Fall (sec)							
		Female				Male			
	Genotype	n=	Mean	Coeff. Variation	SEM	n=	Mean	Coeff. Variation	SEM
4 month	C57BL/6J	16	183.8	26.34%	12.1	16	151.5	28.86%	10.93
	Trem2*R47H	11	154.8	24.06%	11.23	12	142.3	24.86%	10.21
	APOE4	11	139	29.94%	12.54	12	121.9	54.33%	19.11
	APOE4.Trem2*R47H	10	156.4	33.74%	16.69	11	141.5	33.27%	14.19
8 month	C57BL/6J	18	168.8	26.13%	10.4	18	88.89	51.70%	10.83
	Trem2*R47H	12	122	30.94%	10.89	12	123.9	50.16%	17.94
	APOE4	11	143.4	55.85%	24.15	12	84.47	68.54%	16.71
	APOE4.Trem2*R47H	11	128.7	60.97%	23.66	12	88.64	59.80%	15.3
12 month	C57BL/6J	22	118.5	55.26%	13.96	22	56.8	54.53%	6.603
	Trem2*R47H	10	102.7	47.03%	15.28	13	50.1	83.97%	11.67
	APOE4	10	83.3	78.62%	20.71	11	33.12	151.29%	15.11
	APOE4.Trem2*R47H	10	85.63	63.15%	17.1	12	48.11	84.89%	11.79
24 month	C57BL/6J	14	114.9	52.62%	16.16	16	62.69	74.02%	11.6
	Trem2*R47H	13	72.77	87.45%	17.65	13	19.21	107.48%	5.725
	APOE4	10	52.7	76.40%	12.73	11	63.21	86.31%	16.45
	APOE4.Trem2*R47H	7	88.33	45.15%	15.07	8	54.67	95.66%	18.49

Supplemental Table 3. Complete data set of rotarod assay measures of LOAD strains. Individual animals from cross-sectional cohorts housed to the ages indicated were assessed by rotarod assay. Three trials were performed and the average latency to fall is provided. All alleles expressed were homozygous.

		Percent Alternation (%)								Total Arm Entries							
		Female				Male				Female				Male			
	Genotype	n=	Mean	Coeff. Variation	SEM	n=	Mean	Coeff. Variation	SEM	n=	Mean	Coeff. Variation	SEM	n=	Mean	Coeff. Variation	SEM
4 month	C57BL/6J	16	47.34	13.60%	1.085	16	56.18	15.06%	1.592	16	46.5	18.46%	2.26	16	43	20.12%	2.055
	Trem2*R47H	11	49.79	13.32%	1.946	11	53.72	18.66%	2.863	11	45.45	20.10%	2.912	12	38.42	16.76%	1.877
	APOE4	11	48.83	16.15%	2.879	12	52.16	20.94%	2.612	11	42.91	27.44%	3.672	12	42.33	24.13%	2.709
	APOE4.Trem2*R47H	10	45.1	29.90%	3.054	11	54.46	18.01%	2.065	10	47.1	23.25%	2.759	11	41.64	18.61%	2.383
8 month	C57BL/6J	18	51.6	16.97%	2.165	18	56.88	17.81%	1.799	18	40.06	17.53%	1.626	18	42.44	19.35%	2.182
	Trem2*R47H	12	56.18	20.21%	2.543	12	54.88	22.55%	2.714	12	41.67	13.82%	1.437	12	40	18.89%	2.25
	APOE4	12	52.05	17.05%	2.813	12	57.45	10.90%	1.759	12	35.83	29.90%	3.035	12	33.75	25.65%	2.437
	APOE4.Trem2*R47H	12	53.15	16.66%	2.539	12	59.13	25.16%	3.457	12	35.42	18.66%	1.921	12	36.25	18.82%	1.919
12 month	C57BL/6J	21	50.18	18.60%	2.103	22	53.91	20.90%	1.634	21	35.33	28.24%	1.934	22	36.64	24.46%	1.825
	Trem2*R47H	11	58.27	13.32%	3.355	13	58.94	20.18%	2.444	11	38.82	14.00%	1.911	13	32.31	23.09%	2.173
	APOE4	10	47.5	19.07%	2.422	11	50.04	17.65%	3.332	10	35.2	18.39%	1.971	11	36.82	17.96%	2.161
	APOE4.Trem2*R47H	10	53.77	20.99%	2.841	12	55.58	22.55%	3.726	10	40.5	14.80%	1.635	12	33.58	25.89%	2.294
24 month	C57BL/6J	13	58.13	17.74%	2.227	16	56.64	23.28%	2.831	13	33.38	37.52%	3.607	16	31.31	26.83%	1.974
	Trem2*R47H	12	55.59	18.90%	3.042	13	60.72	22.94%	3.271	12	39.25	30.03%	2.66	13	29.92	19.88%	1.666
	APOE4	11	54.53	15.20%	1.788	11	58.09	11.86%	2.35	11	39.91	16.09%	2.095	11	33.36	20.97%	1.918
	APOE4.Trem2*R47H	6	54.7	17.54%	2.927	8	58.1	12.51%	2.777	6	39.17	20.44%	3.06	8	31.38	37.64%	3.751

Supplemental Table 4. Complete data set of spontaneous alternation assay measures of LOAD strains. Individual animals from cross-sectional cohorts housed to the ages indicated were assessed by spontaneous alternation assay in Y-maze. Percentage of successive entries into all three arms in series compared to all arm entries is provided. All alleles expressed were homozygous.

		Sum Distance Traveled (cm; 0-60 min)								Total Sum Vertical Activity (0-60min)								Sum Margin Time (sec)							
		Female				Male				Female				Male				Female				Male			
	Genotype	n=	Mean	Coeff. Variation	SEM	n=	Mean	Coeff. Variation	SEM	n=	Mean	Coeff. Variation	SEM	n=	Mean	Coeff. Variation	SEM	n=	Mean	Coeff. Variation	SEM	n=	Mean	Coeff. Variation	SEM
4 month	C57BL/6J	16	10706	18.93%	506.7	16	10184	24.07%	612.8	16	730.9	27.51%	50.27	16	857.1	30.29%	64.91	16	3128	6.17%	48.27	16	3174	7.34%	58.27
	Trem2*R47H	11	8783	20.58%	545	12	8174	18.83%	444.3	11	537.1	29.73%	48.15	12	719.4	30.75%	63.85	11	3251	4.57%	44.77	12	3128	4.44%	40.12
	APOE4	11	7998	23.29%	561.5	12	8900	14.02%	360.3	11	485.4	37.51%	54.89	12	779.8	20.91%	47.06	11	3155	6.60%	62.81	12	3060	8.80%	77.78
	APOE4. Trem2*R47H	10	8919	11.95%	336.9	11	8562	20.11%	519.1	10	474.2	27.62%	41.42	11	670.3	44.54%	90.02	10	3181	4.20%	42.21	11	3077	5.89%	54.67
8 month	C57BL/6J	18	7822	28.80%	531	18	8971	16.67%	352.6	18	448.8	48.00%	50.78	18	963.9	28.22%	64.11	18	3324	4.34%	33.99	18	3202	4.10%	30.93
	Trem2*R47H	12	8488	28.60%	700.7	12	7756	26.28%	588.4	12	411.2	50.29%	59.69	12	709.8	30.83%	63.18	12	3159	7.55%	68.83	12	3056	6.19%	54.64
	APOE4	12	8286	23.34%	558.2	12	7248	23.19%	485.1	12	458.3	37.34%	49.41	12	710.8	34.75%	71.3	12	3079	6.66%	59.23	12	3080	8.18%	72.76
	APOE4. Trem2*R47H	11	7564	21.71%	495.2	12	7656	20.54%	453.9	11	400.6	46.73%	56.45	12	759.8	23.06%	50.58	11	3218	4.01%	38.9	12	3089	6.81%	60.76
12 month	C57BL/6J	22	7217	35.62%	548.1	21	7391	26.08%	420.7	22	379	62.50%	50.5	21	908	32.23%	63.86	22	3360	5.20%	37.25	21	3101	7.58%	51.29
	Trem2*R47H	11	6971	18.38%	386.3	13	5839	18.11%	293.3	11	372.5	39.26%	44.09	13	553.2	33.82%	51.89	11	3166	7.43%	70.9	13	3027	10.29%	86.34
	APOE4	10	6446	14.89%	303.4	11	6665	33.08%	664.7	10	359.4	38.73%	44.02	11	648	38.23%	74.7	10	3149	6.63%	65.99	11	3113	8.64%	81.13
	APOE4. Trem2*R47H	10	7161	29.92%	677.5	12	6841	37.98%	749.9	10	508.3	42.44%	68.22	12	781.1	50.02%	112.8	10	3271	5.76%	59.6	12	3141	7.78%	70.53
24 month	C57BL/6J	15	6916	32.47%	579.8	16	6616	25.76%	426.1	15	538.1	50.26%	69.84	17	767	51.80%	96.36	15	3143	6.29%	51.06	17	2834	10.52%	72.33
	Trem2*R47H	13	7118	21.23%	419.2	13	5238	23.10%	335.6	6	343.3	31.99%	44.85	13	675.6	33.93%	63.59	13	2945	10.77%	87.96	13	2912	9.70%	78.34
	APOE4	11	7996	32.55%	784.6	11	6614	36.78%	733.5	11	333	37.44%	37.59	11	737.8	37.12%	82.59	11	3007	8.01%	72.65	11	2818	10.19%	86.57
	APOE4. Trem2*R47H	6	7409	32.62%	986.5	8	5846	26.45%	546.6	6	343.5	32.01%	44.88	8	708.9	40.04%	100.3	6	3033	6.32%	78.28	8	2798	18.23%	180.4

Supplemental Table 5. Complete data set of open field assay measures of LOAD strains over time. Individual animals from cross-sectional cohorts housed to the ages indicated were assessed by open field assay. All alleles expressed were homozygous.

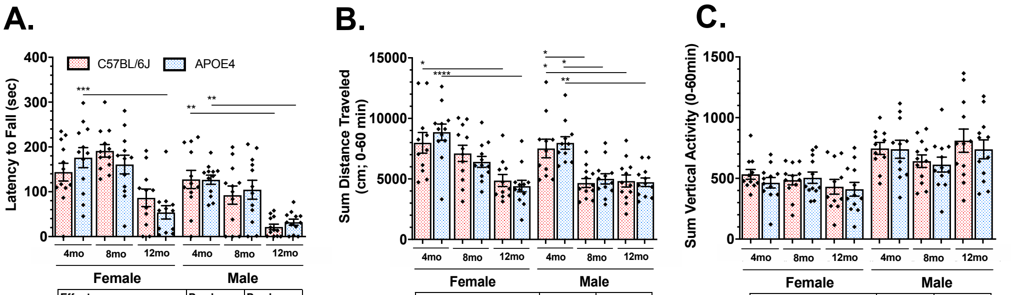

A.

Effect	P value	P value
Age	<0.0001	****
Sex	<0.0001	****
Genotype	0.8811	ns
Age x Sex	0.1812	ns
Age x Genotype	0.4557	ns
Sex x Genotype	0.3685	ns
Age x Sex x Genotype	0.1905	ns

B.

Effect	P value	P value
Age	<0.0001	****
Sex	0.0182	*
Genotype	0.8279	ns
Age x Sex	0.0506	ns
Age x Genotype	0.3873	ns
Sex x Genotype	0.577	ns
Age x Sex x Genotype	0.6205	ns

C.

Effect	P value	P value
Age	0.3795	ns
Sex	<0.0001	****
Genotype	0.4019	ns
Age x Sex	0.0357	*
Age x Genotype	0.8759	ns
Sex x Genotype	0.8672	ns
Age x Sex x Genotype	0.7556	ns

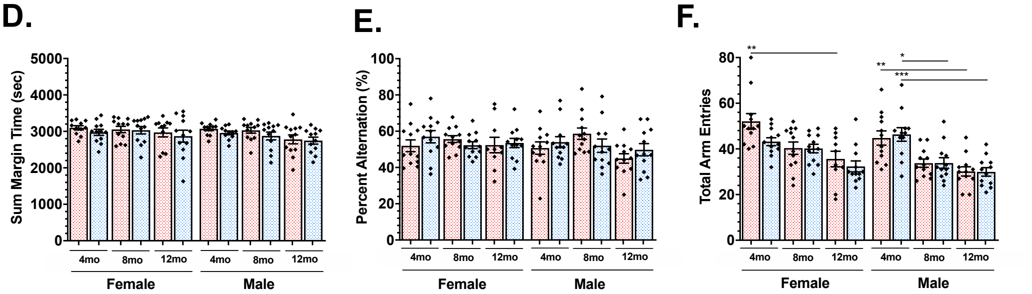

D.

Effect	P value	P value
Age	0.0224	*
Sex	0.1236	ns
Genotype	0.1139	ns
Age x Sex	0.636	ns
Age x Genotype	0.937	ns
Sex x Genotype	0.8703	ns
Age x Sex x Genotype	0.7601	ns

E.

Effect	P value	P value
Age	0.0208	*
Sex	0.0002	***
Genotype	0.2952	ns
Age x Sex	0.6219	ns
Age x Genotype	0.4764	ns
Sex x Genotype	0.3922	ns
Age x Sex x Genotype	0.6469	ns

F.

Effect	P value	P value
Age	0.0208	*
Sex	0.0002	***
Genotype	0.2952	ns
Age x Sex	0.6219	ns
Age x Genotype	0.4764	ns
Sex x Genotype	0.3922	ns
Age x Sex x Genotype	0.6469	ns

Supplemental Figure 2. Expression of human *APOE4* allele does not produce penetrant behavioral phenotype. Littermate-controlled cohorts of young, old, and intermediate ages were tested for differences in neuromuscular coordination (rotarod; A), locomotor activity (open field; B,C), exploratory drive (open field; D), and spatial working memory (spontaneous alternation in Y-maze; E,F) due to age and homozygous expression of humanized *APOE4* allele. Age-dependent differences within genotype and sex determined by three-way ANOVA. Factor effects and effect interaction displayed in tables. *p<0.05; **p<0.01; ***p<0.001.

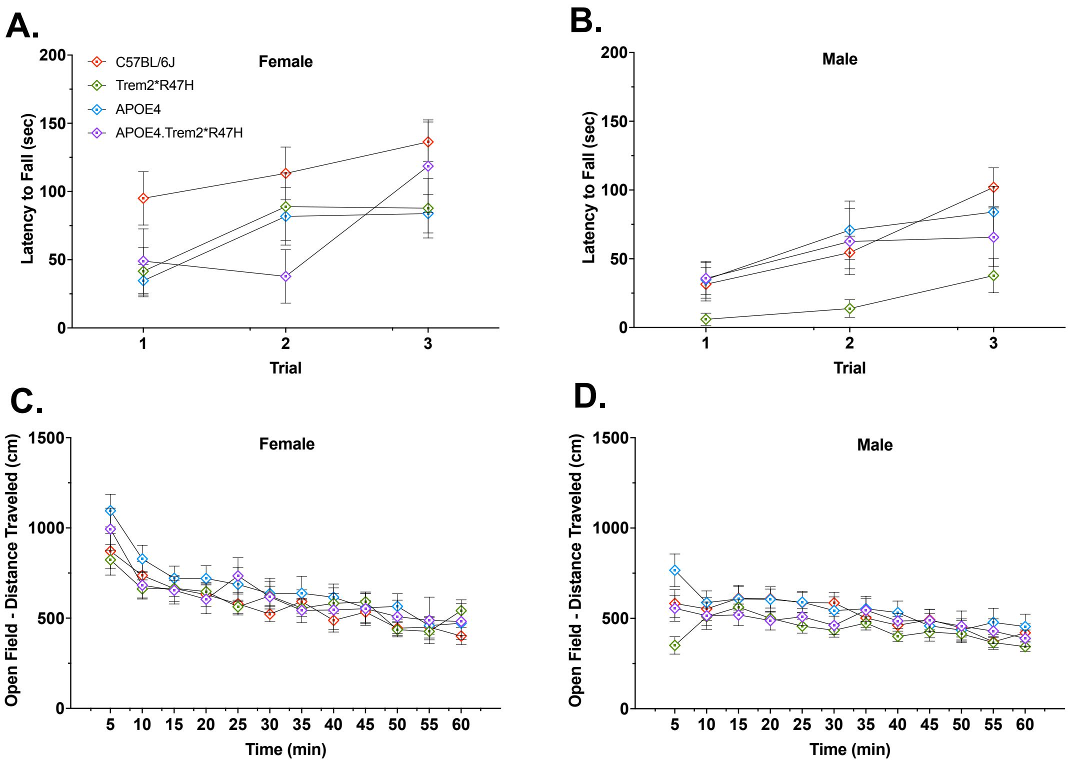

Supplemental Figure 3. Improved performance of locomotor behavioral assays in LOAD mice in successive trials and time bins during testing. Execution of rotarod (A,B) and open field (C,D) behavioral assays by individuals comprising the 24 month aged cohort. Repeated measures in subsequent trials (A,B) and over time (C,D) indicate acclimation of the individual to the testing or environment to determine cognitive plasticity.

A.

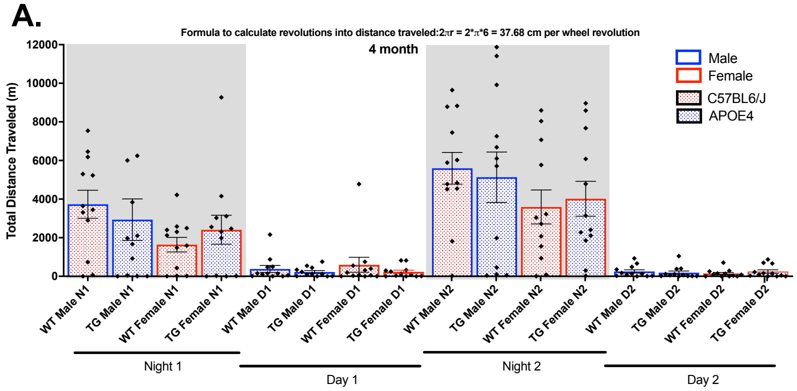

Effect	P value	P value
Age	<0.0001	****
Sex	0.0397	*
Genotype	0.8217	ns
Age x Sex	0.18	ns
Age x Genotype	0.9872	ns
Sex x Genotype	0.3282	ns
Age x Sex x Genotype	0.7454	ns

B.

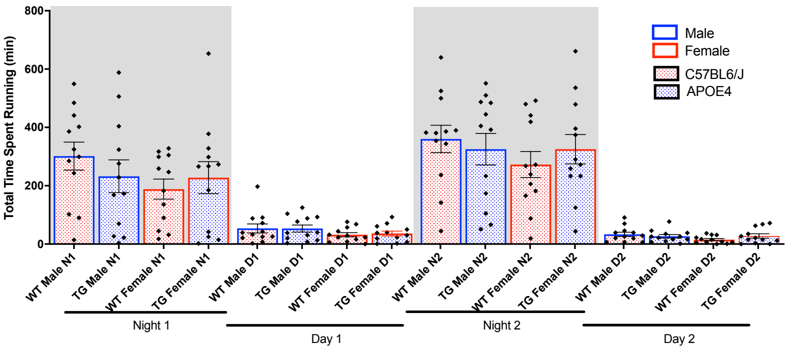

Effect	P value	P value
Age	<0.0001	****
Sex	0.0671	ns
Genotype	0.9854	ns
Age x Sex	0.7389	ns
Age x Genotype	0.9692	ns
Sex x Genotype	0.1213	ns
Age x Sex x Genotype	0.6686	ns

C.

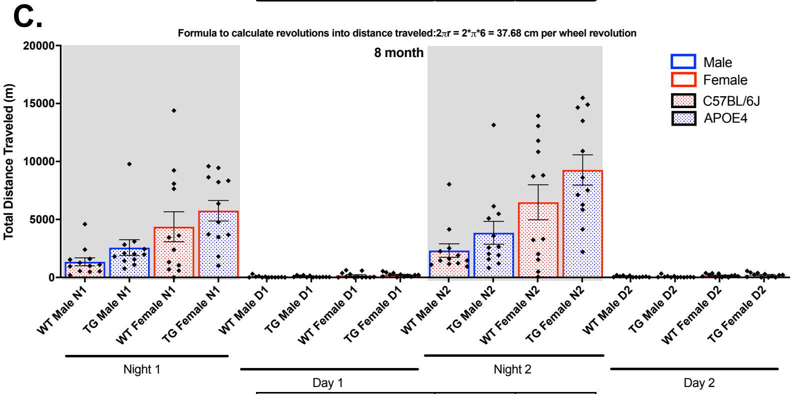

Effect	P value	P value
Age	<0.0001	****
Sex	<0.0001	****
Genotype	0.0009	***
Age x Sex	<0.0001	****
Age x Genotype	0.0884	ns
Sex x Genotype	0.3128	ns
Age x Sex x Genotype	0.8589	ns

D.

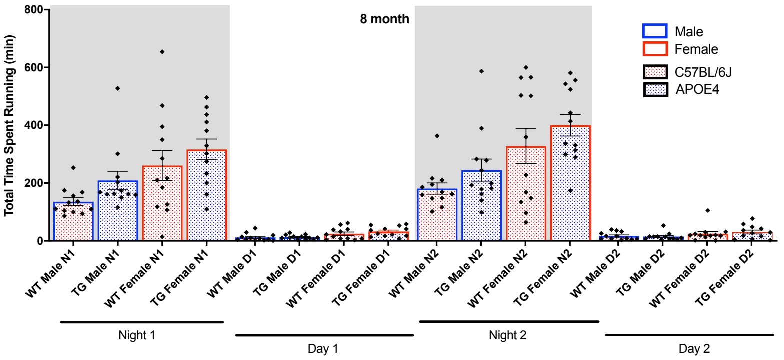

Effect	P value	P value
Age	<0.0001	****
Sex	<0.0001	****
Genotype	0.0015	**
Age x Sex	0.0007	***
Age x Genotype	0.1948	ns
Sex x Genotype	0.7358	ns
Age x Sex x Genotype	0.9749	ns

E.

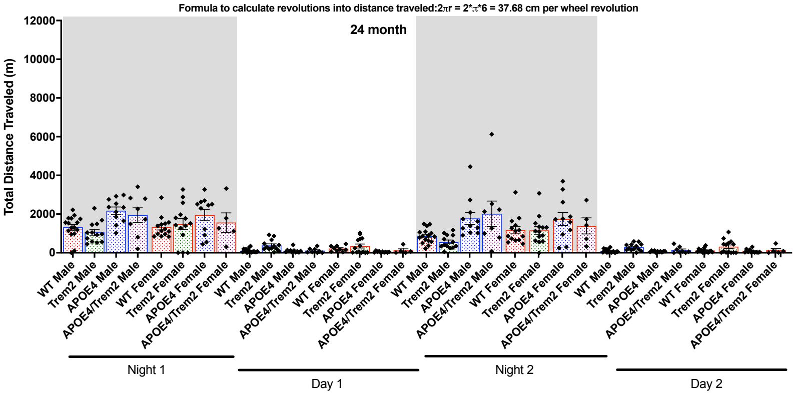

Effect	P value	P value
Age	<0.0001	****
Sex	0.1196	ns
Genotype	0.002	**
Age x Sex	0.3698	ns
Age x Genotype	0.098	ns
Sex x Genotype	0.0133	*
Age x Sex x Genotype	0.2565	ns

F.

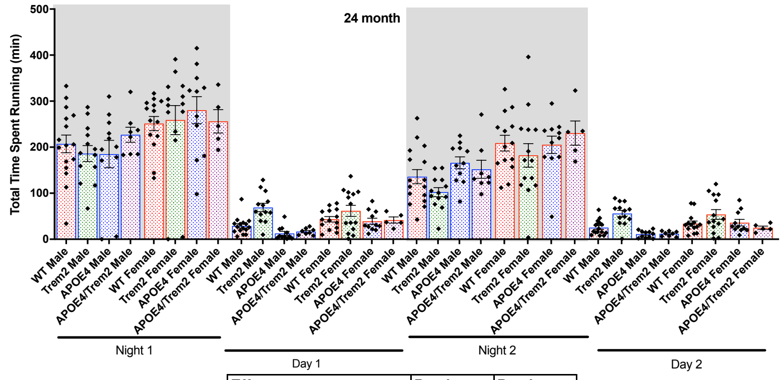

Effect	P value	P value
Age	<0.0001	****
Sex	<0.0001	****
Genotype	0.1699	ns
Agex Sex	0.1047	ns
Age x Genotype	0.2195	ns
Sex x Genotype	0.9603	ns
Age x Sex x Genotype	0.9874	ns

Supplemental Figure 4. Running wheel assay reveals age-dependent changes in animal activity levels. Comparison of activity levels C57BL/6J and B6.*APOE4* mice at 4 (A,B), 8 (C,D), and 24 hours (E,F) by the use of home cage running wheels provided to individually-housed animals over a continuous 48 hour timeline. 12-hour time bins (day vs. night) shows binaural changes in total distance traveled (A, C, E) and time active (B, D, F). C57BL6/J, B6.*APOE4*, B6.*Trem2R47H*, and B6.*APOE4.Trem2R47H* were also assayed together at 24 months of age (E, F) for genotype-driven changes in activity. Factor effects and effect interaction determined by three-way ANOVA displayed in tables. *p<0.05; **p<0.01; ***p<0.001.All alleles expressed were homozygous.

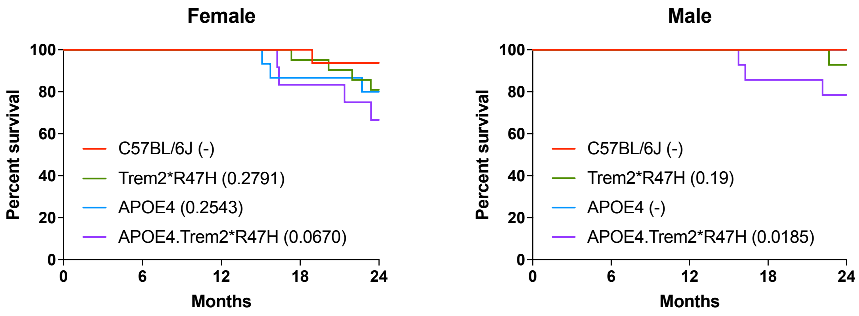

Supplemental Figure 5. *APOE4.Trem2*R47H* mice are more likely to succumb by 24 months of age than C57BL/6J counterparts. Survival of unaffected animals comprising the 24-month cross-sectional cohort. Statistical differences in morbidity determined by log-rank test. All alleles expressed were homozygous.

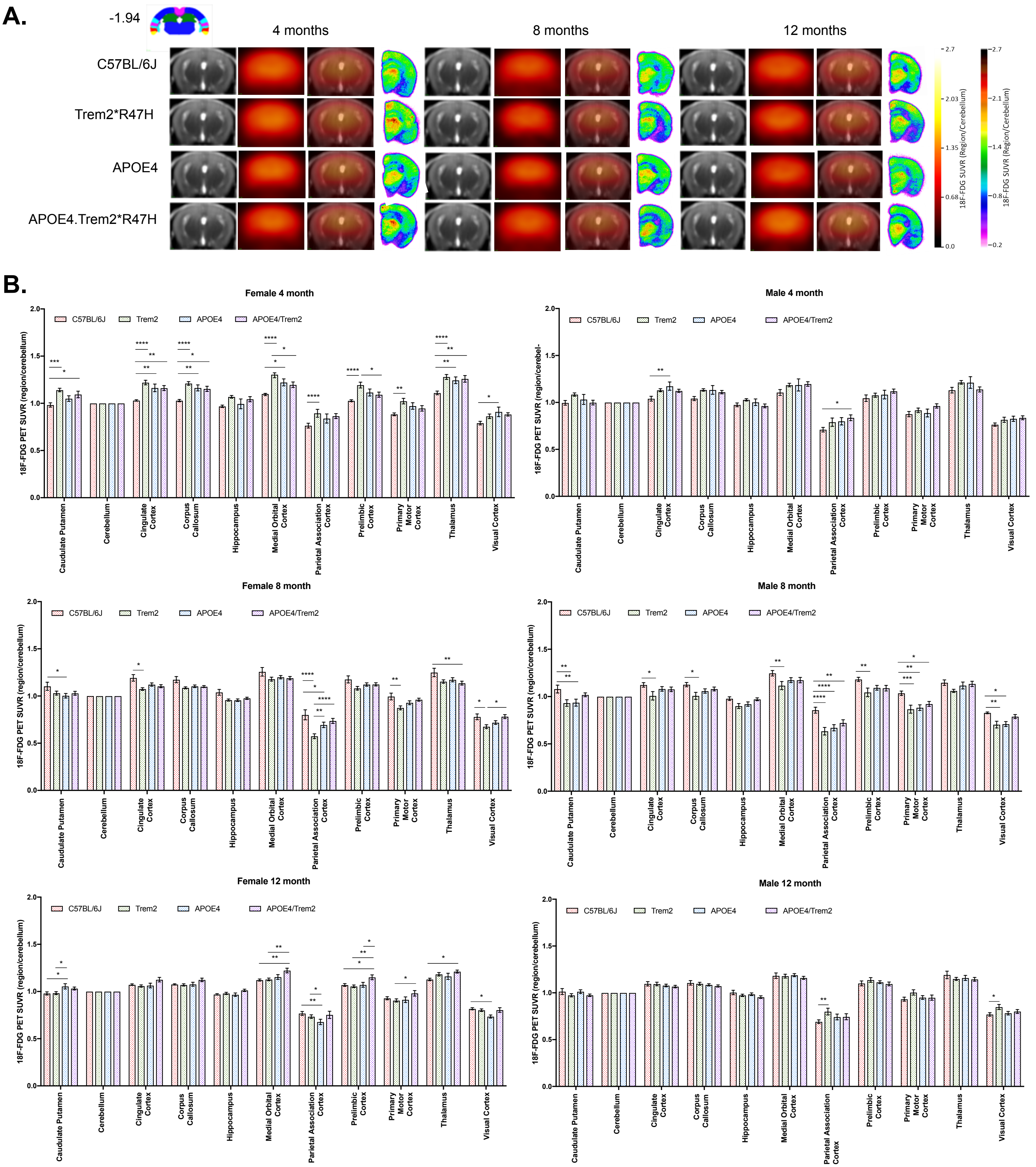

Supplemental Figure 6. Age-related regional changes in glucose metabolism in brains of LOAD mouse strains. Positron emission tomography (PET; red scale) neuroimaging of radioactive 18-FDG marker was used to measure tissue glucose uptake, guided by magnetic resonance imaging (MRI) (black and white) mapping to brain regions of interest, indicated by bregma coordinates (top left) (A). Intensity of PET signal in brains regions, normalized to cerebellum, are quantified in B. Post-mortem autoradiography of coronal brain tissue is represented in A (rainbow; far right). Genotype-dependent differences determined by ANOVA: *p<0.05; **p<0.01; ***p<0.001. All alleles expressed were homozygous.

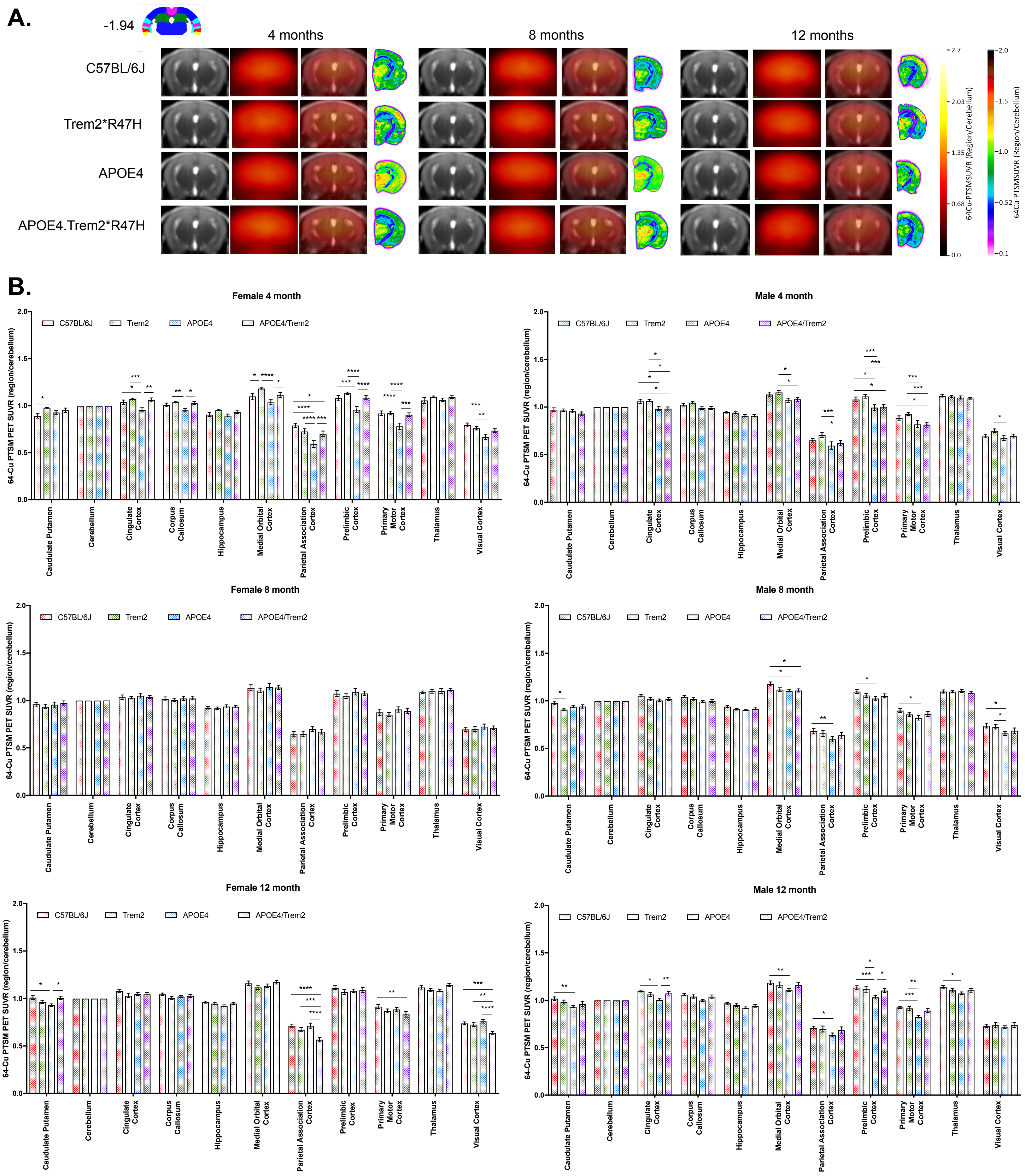

Supplemental Figure 7. Age-related regional changes in perfusion in brains of LOAD mouse strains. Positron emission tomography (PET; red scale) neuroimaging of radioactive 64-Cu marker was used to measure tissue perfusion, guided by magnetic resonance imaging (MRI) (black and white) mapping to brain regions of interest, indicated by bregma coordinates (top left) (A). Intensity of PET signal in brains regions, normalized to cerebellum, are quantified in B. Post-mortem autoradiography of coronal brain tissue is represented in A (rainbow; far right). Genotype-dependent differences determined by ANOVA: *p<0.05; **p<0.01; ***p<0.001. All alleles expressed were homozygous.

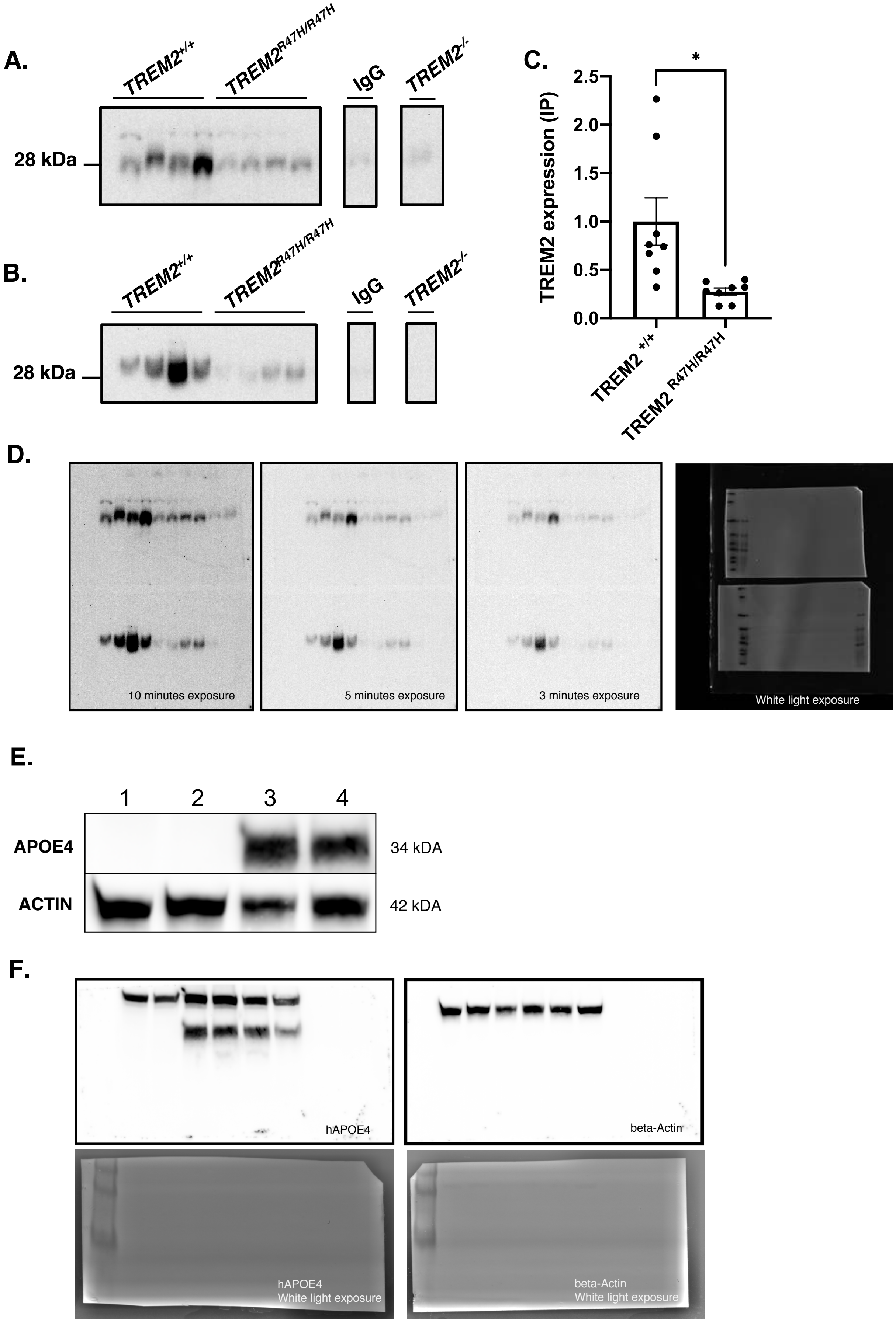

Supplemental Figure 8. Humanized *Trem2*R47H* mutation reduces protein expression of TREM2. Western blot of immunoprecipitated TREM2 from cortical homogenates of wild type (WT) C57BL/6J, B6.*TREM2*$^{+/+}$ B6.*TREM2*$^{R47H/R47H}$, or B6.*TREM2*$^{-/-}$ mice, and IgG as negative control, in duplicate (A, B, D). Quantification of TREM2 intensity relative to WT showing *TREM2*$^{R47H/R47H}$ reduced 72.4% (C), n=8. Student's t-test, * p<0.05. APOE4 levels in whole brain lysates of C57BL/6J (lanes 1 and 2) and B6.*APOE4.Trem2R47H* (lanes 3 and 4) mice, with beta-Actin loading control (E,F).

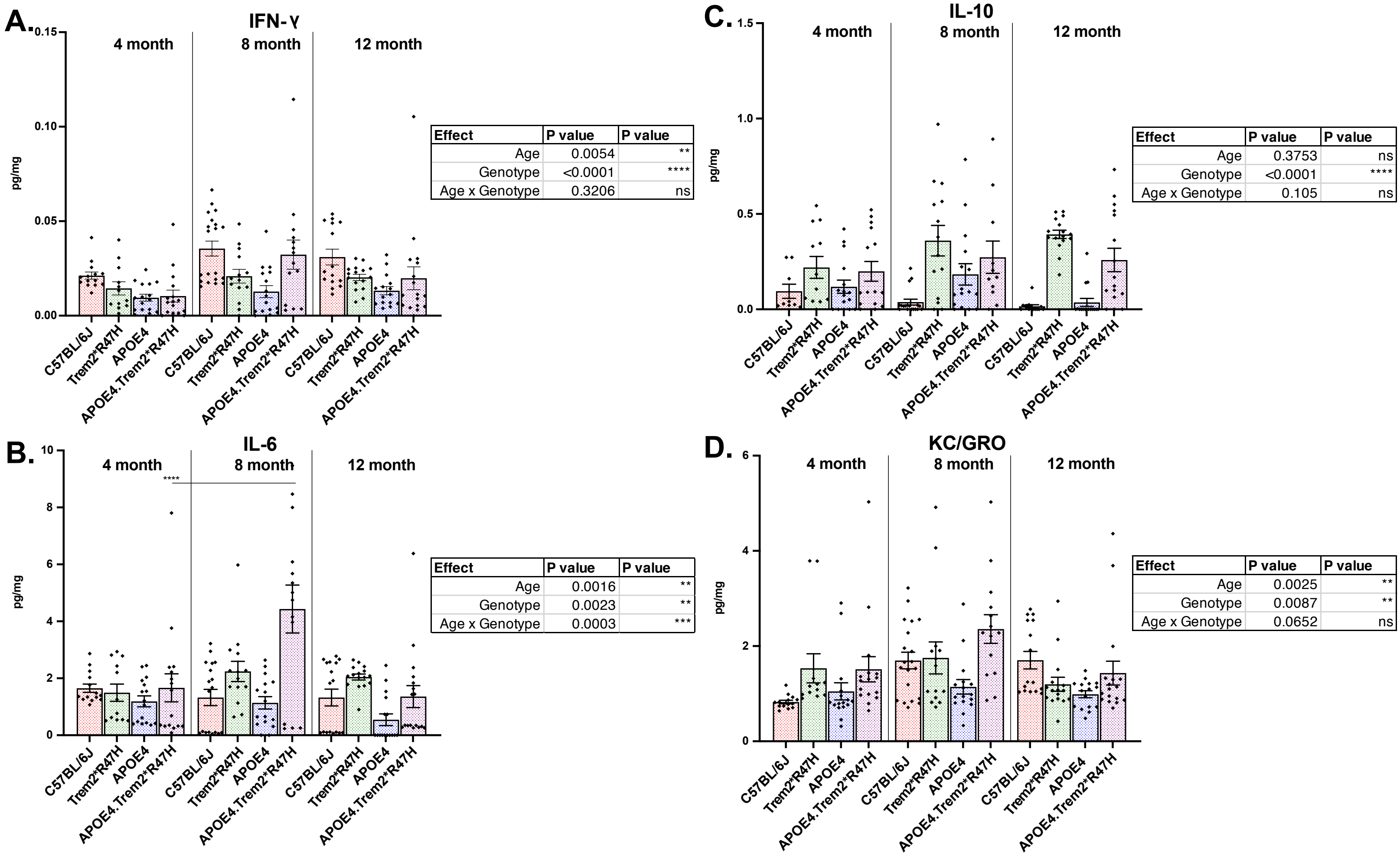

Supplemental Figure 9. Levels of cytokines in brain influenced by both age and genotype. IFN-gamma (IFN-γ), IL-10, IL-6, and KC/GRO levels were measured by multi-plex immunoassays of whole brain lysate (A-D) from untreated animals of all genotypes. Bars represent both male and female data sets. n=12 minimum. Age- and genotype-dependent differences determined by two-way ANOVA. Factor effects and effect interaction displayed in tables. *p<0.05; **p<0.01; ***p<0.001. All alleles expressed were homozygous.

A.

	Differentially Expressed Genes															
	Female								Male							
	4		8		12		24		4		8		12		24	
	Up	Down	Up	Down	Up	Down	Up	Down	Up	Down	Up	Down	Up	Down	Up	Down
**APOE4**	1	2	11	14	0	3	1	23	1	1	42	103	0	1	2	1
**Trem2*R47H**	1	1	2	9	113	172	359	389	5	10	3	29	118	88	15	129
**APOE4.Trem2*R47H**	2	4	2	1	1	4	83	400	1	3	7	32	0	2	24	197

B.

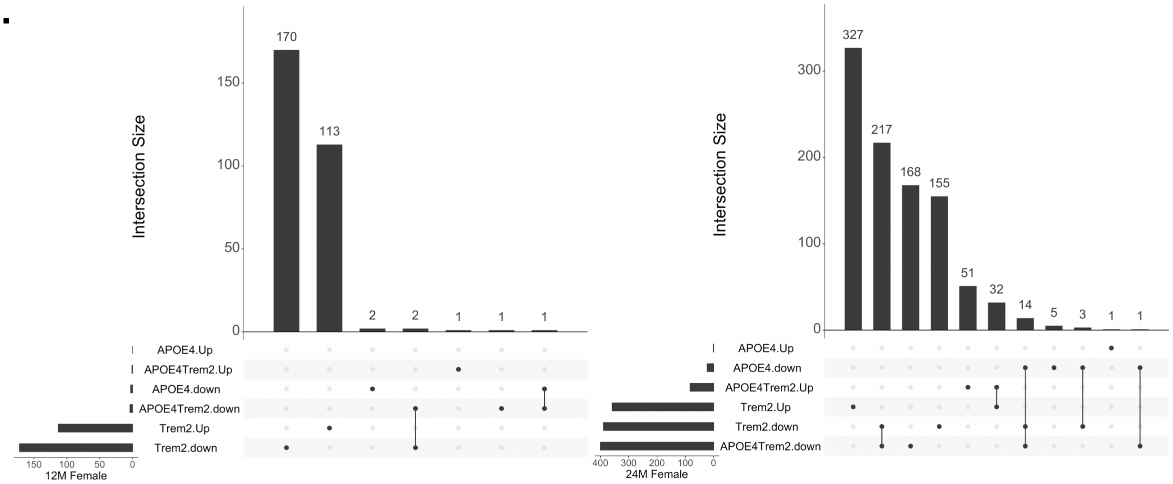

C.

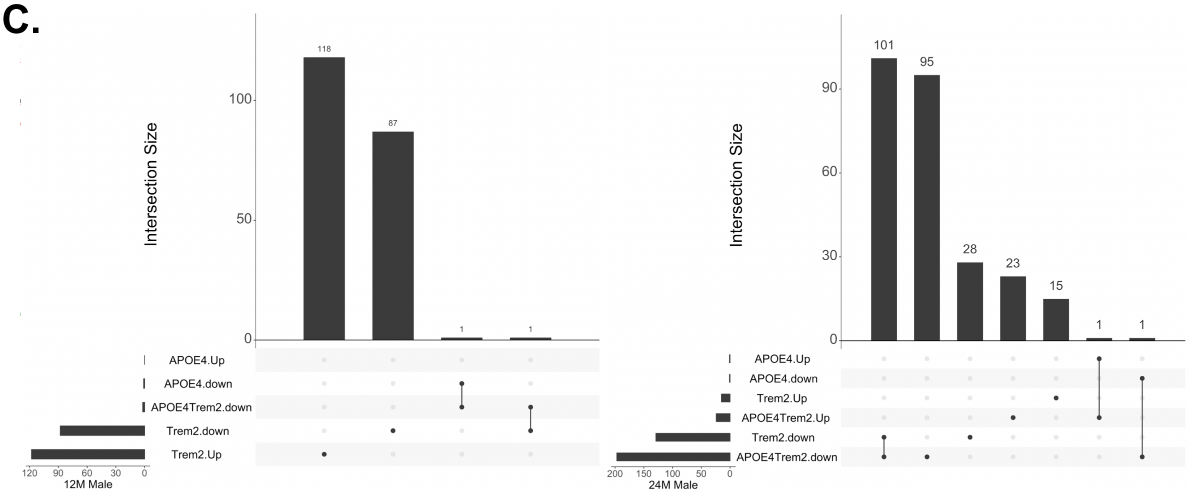

Supplemental Figure 10. Differentially expressed genes in LOAD mice at various ages. Number of differentially expressed genes identified across each mouse model compared to B6 control mice (A). UpsetR plots illustrating the differentially expressed genes shared between genotypes at 12 and 24 months in female (B) and male (C) mice. Abbreviations: UP, Upregulated genes; DOWN, Downregulated genes. All alleles expressed were homozygous.

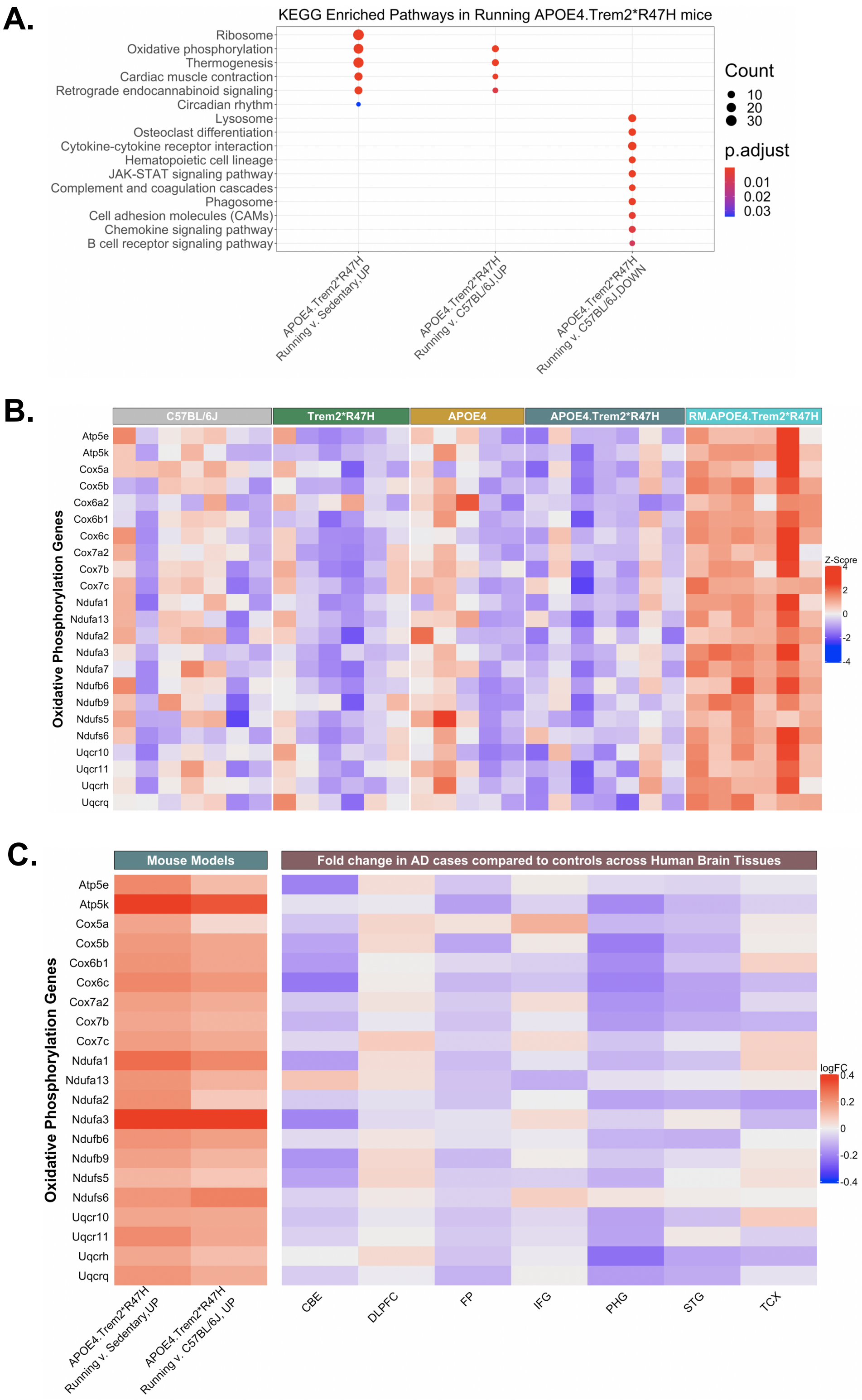

Supplemental Figure 11. Effect of exercise on the mouse brain transcriptome. The KEGG pathway enrichment analysis of the significantly DEG's in the running *APOE4.-Trem2*R47H* mice compare to control (B6) and sedentary *APOE4.Trem2*R47H* mice (A). Heatmap of normalized expression of selected genes associated with oxidative phosphorylation pathways across age and sex-matched mouse models (B). Multiple members of oxidative phosphorylation pathway are downregulated across the seven brain regions from the AMP-AD cohorts (C). All alleles expressed were homozygous.
